# Cardio-oncology from the consulting side: diagnostic thresholds, surveillance and treatment responsibility among cardiologists and internists in a German multicentre survey

**DOI:** 10.1186/s40959-026-00588-7

**Published:** 2026-10-07

**Authors:** Sebastian Poley, Lorena Payo-Anez, Felix M. Heidrich, Karim Ibrahim, Marian Christoph, Tina Haase, Jan Hamann, Lorenz Harst, Silvio Quick

**Affiliations:** 1 Department of Internal Medicine I, Cardiology, Angiology and Intensive Care, Klinikum Chemnitz gGmbH, Medical Campus Chemnitz of the TUD Dresden University of Technology, Dresden, Germany; 2 Department of Internal Medicine and Cardiology, Heart Centre Dresden, Technische Universität Dresden, Dresden, Germany; 3 Centre for Evidence-Based Healthcare, University Hospital Carl Gustav Carus, Technische Universität Dresden, Dresden, Germany

**Keywords:** Cardio-oncology, Cardiotoxicity, Echocardiography, Global longitudinal strain, Cardiovascular pharmacotherapy, Biomarkers, Surveys and questionnaires, Interdisciplinary communication, Hospital-based care

## Abstract

**Background:**

Cancer therapy-related cardiovascular toxicity (CTR-CVT) is a leading non-cancer cause of morbidity and mortality. The 2022 ESC Guidelines define a multidisciplinary pathway in which baseline cardiovascular risk assessment is led by the treating oncology or haematology team, with cardiological involvement guided by the resulting risk; how this is put into practice in hospitals is poorly characterised. Previous surveys have described the treating specialties; we examined the receiving cardiologists and internists.

**Methods:**

Cross-sectional online survey of physicians in cardiology, internal medicine, gynaecology and oncology at 1,435 German hospital departments (February–October 2025). This pre-specified analysis addressed cardiological involvement in the pathway and in risk stratification, left-ventricular ejection fraction (LVEF)-decline thresholds, strain use and biomarker testing in a standardised high-risk scenario (a patient with pre-existing heart failure receiving anthracycline chemotherapy). Findings are self-reported; comparisons exploratory.

**Results:**

One hundred twenty physicians responded (44 cardiologists, 28 oncologists, 27 gynaecologists, 21 internists; 73.3% academic or university hospitals). Risk stratification showed the lowest cardiological involvement (42.9%, 15/35); 51.0% (26/51) of cardiologists and internists performed it themselves or within a team, and 19.6% (10/51) regarded it as the treating oncologist’s task. LVEF-decline thresholds were asked in two non-equivalent versions and are reported separately. Any LVEF decline was regarded as cardiotoxicity by 38.2% (13/34) of cardiologists and 61.5% (8/13) of internists, and by 26.7% (4/15) of gynaecologists and 39.1% (9/23) of oncologists; a decline exceeding 10% points by 55.9% and 23.1%, respectively. Routine strain use was reported by 76.5% (26/34) of cardiologists and 36.4% (4/11) of internists. Twelve-month biomarker testing in this scenario was infrequent even in cardiology (20.6%).

**Conclusions:**

Among these predominantly academic physicians, risk stratification had the least clearly assigned ownership, the LVEF decline regarded as relevant varied widely within each version, routine strain use differed between cardiology and internal medicine, and late post-treatment biomarker testing was infrequent even in cardiology. These hypothesis-generating findings identify targets—assigned responsibility for risk stratification, explicit LVEF thresholds, implementation of routine strain imaging and structured post-treatment surveillance—whose effect on CTR-CVT outcomes requires prospective testing.

**Supplementary Information:**

The online version contains supplementary material available at https://doi.org/10.1186/s40959-026-00588-7.

## Background

Modern anticancer therapy has markedly improved survival, but many antineoplastic agents carry a substantial risk of cancer therapy-related cardiovascular toxicity (CTR-CVT), now a leading non-cancer cause of morbidity and mortality in oncology patients [1, 2]. The spectrum ranges from asymptomatic left-ventricular dysfunction to overt heart failure, arrhythmia, myocarditis and vascular events [3], and its prevention depends not only on identifying patients at risk but on the downstream cardiological tasks of surveillance and treatment.

The 2022 European Society of Cardiology (ESC) Guidelines on cardio-oncology provide the first comprehensive framework for this pathway, integrating baseline risk stratification with the Heart Failure Association–International Cardio-Oncology Society (HFA-ICOS) tool, serial cardiac imaging and biomarkers, and the use of cardioprotective and heart-failure pharmacotherapy [4, 5]. Two elements of this framework depend heavily on cardiological expertise. First, echocardiographic surveillance combines left-ventricular ejection fraction (LVEF) with global longitudinal strain (GLS); in the SUCCOUR trial a GLS-guided strategy was associated with fewer cardiotoxicity events at one year than an ejection-fraction-guided strategy, although its primary endpoint (change in LVEF) did not differ significantly and the event difference was not sustained at three years [6]. Second, cardioprotective and heart-failure pharmacotherapy requires cardiological judgement on who is treated, with what, and when [7–9].

Whereas the guidelines and most implementation surveys have focused on the treating oncological specialties—the “demand” side of cardio-oncology—comparatively little is known about how the cardiologists and internists who receive these referrals actually practise: which left-ventricular ejection fraction (LVEF) thresholds they treat as relevant, whether they use strain imaging, how they conduct biomarker surveillance in a standardised high-risk scenario (a patient with pre-existing heart failure receiving anthracycline chemotherapy), which cardioprotective agents they know and use, and who they believe is responsible for initiating that treatment. An earlier survey by the ESC Council of Cardio-Oncology and the Council for Cardiology Practice addressed this side of the pathway from the perspective of self-assessed competence, finding that most responding cardiologists rated their skills in managing cancer therapy-related cardiovascular conditions as no better than fair and that the large majority regarded cardio-oncology as a field in need of substantially more education [10]. These questions determine whether a correctly risk-stratified patient subsequently receives adequate surveillance and protection.

This study is part of a pre-planned programme of work on interdisciplinary cardio-oncology in Germany. In a companion outpatient study we characterised the ambulatory management of cardiotoxicity [11], and in a companion report from the present hospital survey we described baseline risk stratification, cardiac biomarker use, cardio-oncology boards and interdisciplinary communication, chiefly among treating gynaecologists and oncologists [12].

## Methods

### Study design and population

This cross-sectional, multicentre survey was conducted among hospital-based physicians in Germany. Departments in the four specialties routinely involved in the care of patients receiving potentially cardiotoxic chemotherapy — cardiology, internal medicine, gynaecology and oncology — were identified across multiple German federal states through a systematic search of publicly available contact information, and one physician was identified and invited per department. Eligibility was thus defined at the level of specialty; whether an individual department in fact treats patients receiving such chemotherapy could not be established from publicly available sources and was not verified before invitation. Between February and October 2025, 1,435 individual departmental physician contacts were approached by e-mail to complete an anonymous online survey, and 120 completed questionnaires were returned, corresponding to a response rate of 8.4%. This figure underestimates the response rate among physicians who were both eligible and reached, for two reasons: successful delivery could not be verified owing to institutional spam filtering, and the sampling frame was not pre-screened for eligibility, so an unknown proportion of invitations will have reached departments to which the questionnaire did not apply. Participation was strictly anonymous; respondents were not asked to disclose their institution or federal state. The invitation and the questionnaire both described the survey as concerning inpatient cardio-oncology care, so respondents answered within that frame; no item, however, restricted answers to a defined inpatient episode, and respondents were asked to estimate the proportion of their patients managed as outpatients. The findings therefore describe the practice of hospital-based physicians, a proportion of whose clinical work is ambulatory, rather than inpatient care as such. The responses were not verified against records, and no patient-level care or outcomes were assessed.

### Survey administration

The survey was hosted on SoSci Survey. Skip logic was applied, so that follow-up items were displayed only when applicable. Because participation was anonymous, responses could not be linked across sessions or de-duplicated at the respondent level.

### Questionnaire and scope of the present analysis

The questionnaire was developed by a multidisciplinary team of cardiologists, oncologists and health-services researchers, covered nine thematic domains, and was pilot-tested with seven hospital-based physicians before distribution. Multidisciplinary development and pilot-testing supported the face validity of the instrument but did not formally establish content validity; because the instrument assessed heterogeneous clinical workflows rather than a single latent construct, formal internal-consistency testing (e.g. Cronbach’s alpha) was not applicable. The risk-factor item did not reproduce the HFA-ICOS baseline risk proforma in full; it presented a selection of factors drawn from it, chosen for clinical familiarity and brevity, and does not include all items of the proforma. Responses to it are therefore not interchangeable with an HFA-ICOS risk category. The questionnaire was administered in German; the German original and an English translation are provided as Additional file 1.

To avoid duplication with the companion report from the same hospital-based survey [12]—which analysed baseline risk stratification, surveillance practices, cardio-oncology boards and general communication—the present analysis was restricted to a separate, pre-specified set of items. These concerned the contribution of cardiology and internal medicine to the treatment pathway and to the cardiac work-up; echocardiographic and strain-based surveillance and the thresholds at which cardiotoxicity is flagged; biomarker testing in a standardised high-risk scenario (anthracycline chemotherapy with pre-existing heart failure); the familiarity with, use of, and initiation of cardiovascular drugs; and interdisciplinary communication and satisfaction with cardio-oncological care. Items were multiple-choice, Likert-scale or free-text numeric entries. For items that permitted multiple responses, percentages denote the proportion of responding physicians who selected an option and may therefore sum to more than 100%. The allocation of survey items between the two hospital-based analyses was defined before analysis, and a mapping of the survey items to their analyses, and to the primary or secondary character of that analysis, is provided in Additional file 2. To allow the present manuscript to stand alone, essential participant characteristics are reproduced in the Results and the interpretation of our findings does not rely on unpublished results from the companion report [12].

Two features of the instrument require explicit statement. First, the item on echocardiographic thresholds was administered in two non-equivalent versions: cardiologists and internists were asked at which LVEF decline to a value below the reference limits they assume relevant cardiotoxicity and communicate it, whereas gynaecologists and oncologists were asked the same question without the requirement that the resulting value fall below the reference limits. The two versions do not describe the same clinical event; responses are therefore reported separately by version and are never compared across versions. The item also did not specify whether the decline was absolute or relative, and did not capture the global longitudinal strain and biomarker components of the guideline definition of CTR-CVT; it records the threshold at which respondents report cardiotoxicity and cannot measure concordance with that definition. Second, two items presented a standardised high-risk scenario — a patient with known heart failure receiving anthracycline chemotherapy — and asked at which time points such a patient would be seen and at which cardiac biomarkers would be measured. The scenario was deliberately confined to a single dominant risk feature: in the HFA-ICOS baseline risk proforma, pre-existing heart failure or cardiomyopathy is by itself sufficient for the highest risk categories in patients receiving anthracyclines, and the baseline assessment and surveillance schedule recommended for the high- and very-high-risk categories does not differ for these regimens, so further risk features would have lengthened the item without changing what the scenario asks of respondents. It is not an operationalisation of the HFA-ICOS categories and represents only one of several routes into a high-risk classification.

### Statistical analysis

Responses are summarised descriptively. Categorical variables are presented as counts and percentages, continuous variables as medians with interquartile ranges (IQR) or ranges, as indicated. Throughout, “valid percent” denotes the percentage among respondents who answered the respective item (the valid denominator), excluding item non-response and questions not displayed by skip logic. Numerators and valid denominators are reported in the text for key findings; tables and figures present valid percentages with the corresponding group denominators in their legends. Missing values arose from three sources—branching (skip) logic that displayed follow-up items only when applicable, optional items, and item non-response—and were not imputed; an item-level account of denominators and missing data is provided in Additional file 3 (Table S2). For every table and figure, the number of respondents eligible to receive the item, the number who answered it, the number excluded by skip logic and the number lost to item non-response are reported in Additional file 3 (Table S2).

Because this was an exploratory, descriptive survey, no single primary outcome was defined and no a priori power calculation was performed; the achieved sample size was determined by the number of respondents. Where groups are compared, Fisher’s exact test (two groups) and the Fisher–Freeman–Halton exact test (more than two groups) were used, the latter in preference to the chi-square test because several cells had expected counts below five. All inferential analyses are exploratory: no correction for multiple testing was applied, p-values are reported descriptively rather than to establish significance, and findings based on small subgroups are hypothesis-generating and may be sensitive to a change in one or two responses. Cardiology and internal medicine, and oncology and gynaecology, are analysed and displayed as separate specialties; where a combined estimate is shown it is descriptive only and does not imply that these specialties are equivalent in training, case-mix or prescribing authority. Analyses used SPSS (IBM Corp., Armonk, NY, USA). A large language model was used solely for language editing; it was not used for data generation or scientific interpretation.

## Results

### Participant characteristics and care setting

A total of 120 physicians responded: 44 cardiologists (36.7%), 28 oncologists (23.3%), 27 gynaecologists (22.5%) and 21 internists (17.5%). Most worked at academic teaching hospitals (54, 45.0%) or university hospitals (34, 28.3%), with fewer at community hospitals (22, 18.3%), maximum-care hospitals (9, 7.5%) and one university hospital with a dedicated cardio-oncology unit (1, 0.8%); academic teaching and university hospitals thus accounted for 73.3% of respondents (88/120). Cardiologists and internists reported a median of 5 chemotherapy patients per month referred for cardiac evaluation (range 0–100), whereas gynaecologists and oncologists treated a median of 30 such patients monthly (range 0–500).

### Involvement of cardiology and internal medicine across the treatment pathway

Cardiologists and internists were asked at which steps of the potentially cardiotoxic treatment pathway they were actively involved (Additional file 3: Fig. S1). Involvement was concentrated in the peri-therapeutic diagnostic phase and was consistently higher for cardiologists than for internists. Cardiologists reported the greatest involvement in cardiac diagnostics during and after chemotherapy (80.0% each), cardiac diagnostics before chemotherapy (74.3%) and in initiating treatment once cardiotoxicity had occurred (71.4%), whereas internists were involved less than half as often in the latter step (23.5%). Formal cardiotoxicity risk stratification was the step of chemotherapy monitoring and therapy management in which cardiologists were least involved (42.9%). Internists were comparatively more often represented in tumour boards (52.9% vs. 14.3%), while cardiologists were more strongly embedded in longitudinal cardiac surveillance.

### Risk stratification from the cardiology and internal-medicine perspective

When cardiologists and internists were asked whether they themselves performed stratification of the potential cardiotoxic risk (*n* = 51), only about half did so: 41.2% within an interdisciplinary team and a further 9.8% independently, whereas 29.4% did not perform it at all and 19.6% regarded it as generally the responsibility of the treating oncologist or haematologist. The pattern was similar for cardiologists (in-team 40.0%, alone 11.4%, not performed 28.6%, oncologist’s task 20.0%) and internists (in-team 43.8%, alone 6.3%, not performed 31.3%, oncologist’s task 18.8%). Where a risk assessment was performed, respondents overwhelmingly reported that it influenced oncological decision-making through a shared rather than a unilateral process: 88.5% stated that the assessment had an influence and was translated into a joint evaluation via consultation with the oncologist or gynaecologist, while only 7.7% issued a unilateral therapeutic recommendation and 3.8% reported that it had no influence and was not communicated.

### Cardiac work-up requested from cardiology by the treating specialties

Gynaecologists and oncologists were asked which investigations they specifically requested from cardiology (Table 1). Oncologists requested a broader panel than gynaecologists (median 3 [IQR 2–4.5] vs. 2.5 [IQR 2–4] distinct investigations). Echocardiography and electrocardiography (ECG) were the most frequently requested modalities in both groups (echocardiography 95.8% in oncology vs. 85.0% in gynaecology). The largest between-specialty gaps were seen for the quantitative and biomarker-based elements of the work-up: ergometry (45.8% vs. 20.0%), troponin (29.2% vs. 10.0%), natriuretic peptides (37.5% vs. 20.0%) and additional laboratory parameters (25.0% vs. 10.0%) were each requested roughly twice as often by oncologists. These figures reflect investigations that respondents reported requesting from cardiology rather than investigations verified as performed, and the gynaecology–oncology differences were not formally tested; they are presented as observed proportions (gynaecology *n* = 20, oncology *n* = 24).

**Table 1:** Investigations requested from cardiology by gynaecology and oncology

Requested investigation	Gynaecology	Oncology
History	35.0	37.5
ECG	75.0	70.8
Echocardiography	85.0	95.8
Ergometry	20.0	45.8
Troponin	10.0	29.2
Natriuretic peptide (BNP/NT-proBNP)	20.0	37.5
Additional laboratory parameters (glucose, GFR, lipids)	10.0	25.0
Other	10.0	8.3

Values are valid percent of responding physicians who reported requesting each investigation from cardiology, not of patients examined (gynaecology 20 of 27 eligible respondents, oncology 24 of 28; no respondent was excluded by skip logic); multiple responses were possible

*BNP *B-type natriuretic peptide,* ECG *electrocardiography,* GFR *glomerular filtration rate,* NT-proBNP *N-terminal pro-B-type natriuretic peptide

### Echocardiographic thresholds for assuming relevant cardiotoxicity

**Fig. 1:**
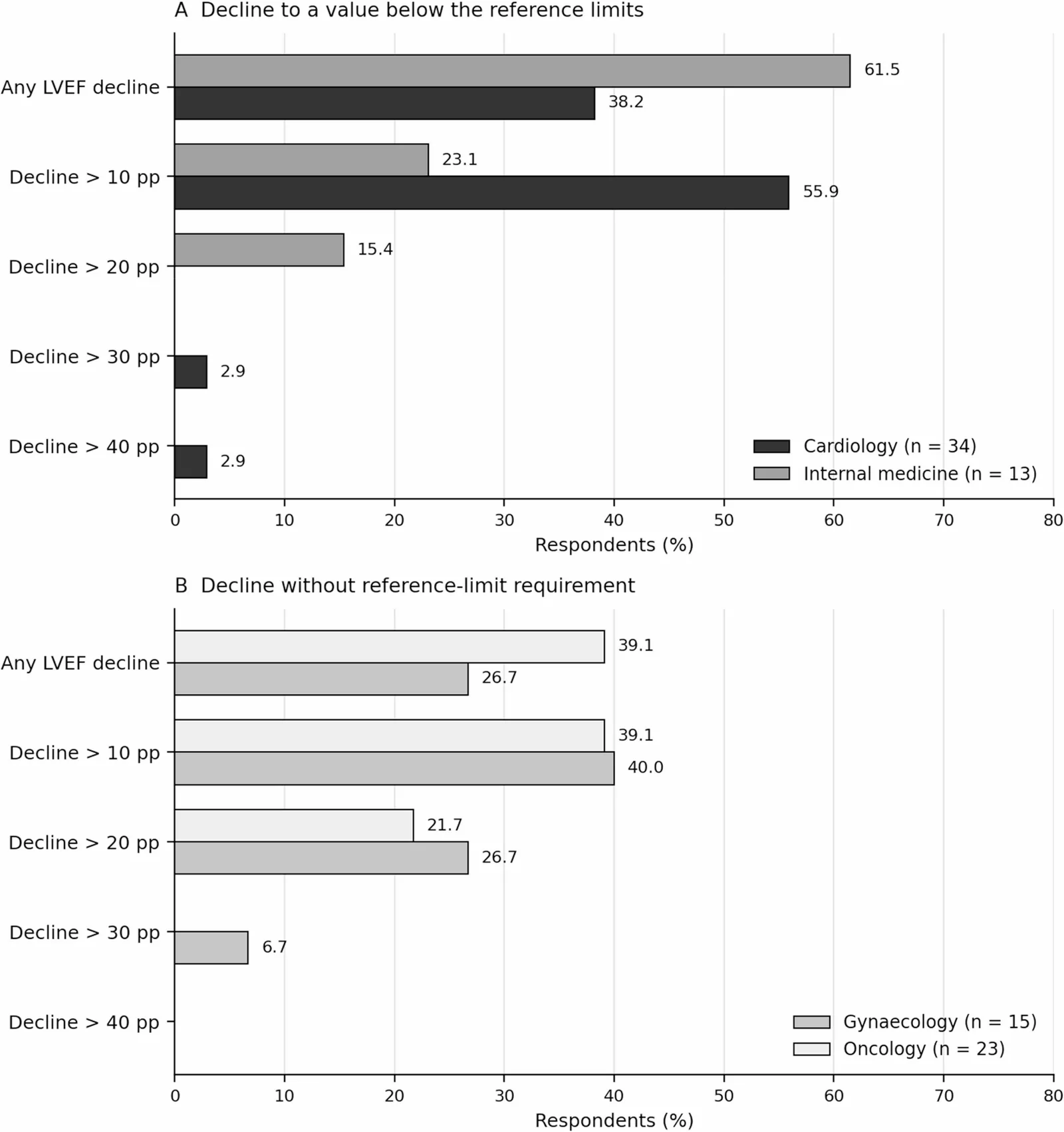
Self-reported echocardiographic thresholds for relevant cardiotoxicity, by specialty and questionnaire version. **A** Cardiology (*n* = 34) and internal medicine (*n* = 13), asked about a decline to a value below the reference limits. **B** Gynaecology (*n* = 15) and oncology (*n* = 23), asked without that requirement. Panels **A** and **B** are not comparable with one another. Of 65 eligible respondents in cardiology and internal medicine, 47 answered the item (**A**); of 55 in gynaecology and oncology, 38 answered (**B**); no respondent was excluded by skip logic

The threshold at which a decline in LVEF was regarded as relevant cardiotoxicity was asked in two versions. Cardiologists and internists were asked at which decline to a value below the reference limits they assume relevant cardiotoxicity and communicate it; gynaecologists and oncologists were asked the same question without the requirement that the resulting value fall below the reference limits. The two versions do not describe the same clinical event and are reported separately throughout (Fig. 1).

Among cardiologists (*n* = 34), 55.9% (19) selected a decline of more than 10% points, 38.2% (13) regarded any LVEF decline as cardiotoxicity, and 2.9% (1) each selected thresholds of more than 30 and more than 40% points; no cardiologist selected more than 20% points. Among internists (*n* = 13), 23.1% (3) selected more than 10% points, 61.5% (8) regarded any decline as cardiotoxicity and 15.4% (2) selected more than 20% points.

Among gynaecologists (*n* = 15), 40.0% (6) selected more than 10% points, 26.7% (4) any decline, 26.7% (4) more than 20 and 6.7% (1) more than 30% points. Among oncologists (*n* = 23), 39.1% (9) each selected more than 10% points and any decline, and 21.7% (5) more than 20% points.

Responses were thus distributed across all available options in every specialty. In both versions a substantial proportion selected self-reported response rules that do not match the full ESC definition, either because they lie above every threshold defined in it or because any decline, including one within the normal range, was treated as cardiotoxicity.

### Myocardial strain imaging: adoption and barriers

Beyond LVEF, the routine use of echocardiographic strain for follow-up after chemotherapy differed between the consulting disciplines: 76.5% of cardiologists (26/34) but 36.4% of internists (4/11) reported using strain routinely (Fisher’s exact *p* = 0.026, exploratory). Among the few respondents who did not use strain—only 8 cardiologists and 7 internists—the barriers cited differed, with internists more often reporting a lack of expertise and insufficient acoustic windows and cardiologists more often reporting time and personnel constraints (Additional file 3: Table S1). These barrier counts are too small to establish distinct specialty-specific mechanisms and are reported descriptively only.

### Biomarker surveillance in the standardised high-risk scenario, cardiology and internal medicine

For the standardised high-risk scenario introduced in the Methods (a patient receiving anthracycline chemotherapy with pre-existing heart failure), cardiologists and internists were asked how frequently they determined N-terminal pro-B-type natriuretic peptide (NT-proBNP) or troponin (Table 2). Cardiologists (*n* = 34) reported the most complete baseline testing (before chemotherapy 64.7%; after each cycle 38.2%; after completion 38.2%), but late testing remained infrequent even in this group (6 months 11.8%, 12 months 20.6%), and acute cardiac events remained a common trigger (47.1%). Internists tested less often at each proactive time point (before chemotherapy 36.4%, after each cycle 27.3%, after completion 27.3%) and reported no testing at 6 or 12 months, while 18.2% determined no biomarkers at all. Reasons given for not testing—chiefly that it was regarded as the oncologist’s task—rested on very small subgroups (internal medicine *n* = 2, cardiology *n* = 4) and are not interpreted further. The same pattern was evident in our earlier survey of the ambulatory setting, where cardiac biomarkers were determined by only a minority of respondents [11], suggesting that this is not specific to hospital-based practice.

**Table 2:** Timing of biomarker testing in the high-risk scenario, cardiology versus internal medicine

Time point / trigger	Cardiology	Internal medicine
Before chemotherapy	64.7	36.4
After each cycle	38.2	27.3
After every 2nd cycle	20.6	9.1
After completion	38.2	27.3
3 months post-therapy	17.6	9.1
6 months post-therapy	11.8	0
12 months post-therapy	20.6	0
Acute cardiac symptoms	47.1	45.5
No biomarkers measured	11.8	18.2
Other	17.6	27.3

Values are valid percent for the high-risk scenario (a patient receiving anthracycline chemotherapy with pre-existing heart failure); cardiology *n* = 34, internal medicine *n* = 11. Multiple responses were possible. Values are proportions of responding physicians reporting the respective time point, not of patients tested. Of 65 eligible respondents in cardiology and internal medicine, 45 answered the item and none were excluded by skip logic

### Familiarity with and use of cardiovascular drugs

This item asked whether respondents “knew and used”, “knew but did not use”, or neither knew nor used “cardioprotective substances”, without defining the drugs, distinguishing primary prophylaxis from treatment of established dysfunction, or separating cardiovascular indications; the accompanying list combined established neurohormonal heart-failure medications with agents of only potential cardioprotective value, such as statins. The item therefore does not measure guideline-defined cardioprotection, and its results are reported only as familiarity with, and use of, a heterogeneous list of cardiovascular drugs. With this caveat, the distribution across the three response options differed by specialty (Additional file 3: Fig. S4): “knows and uses” was reported by 56.5% of oncologists, 48.5% of cardiologists, 36.4% of internists and 6.3% of gynaecologists; “knows but does not use” by 39.1%, 21.2%, 9.1% and 43.8%, respectively; and “neither” by 4.3%, 30.3%, 54.5% and 50.0%. No inferential comparison is drawn from this item, and it cannot support conclusions about an awareness–action gap, about underuse of guideline-defined cardioprotection, or about the delivery of primary prevention.

Among item-specific valid responders, the agents most frequently reported were neurohormonal heart-failure medications (Additional file 3: Table S3). ACE inhibitors and beta-blockers were the agents most frequently reported in every specialty, followed by mineralocorticoid-receptor antagonists (MRA) and statins; angiotensin receptor–neprilysin inhibitors (ARNI) were reported by cardiology and internal medicine, this option not having been offered to gynaecology or oncology. Because the response options differed between specialties, that table should not be read as a strict like-for-like comparison; the internal-medicine and gynaecology columns rest on four respondents each, and the item is therefore reported in the supplement only. Antiplatelet agents, anticoagulants and iron chelators were offered as response options but are not reported: the first two are not cardioprotective with respect to cancer therapy-related cardiac dysfunction, and the item wording did not distinguish this narrow sense of cardioprotection from the broader prevention of cardiovascular complications of cancer therapy. Between-specialty comparison of that table is not valid for any agent.

### Who initiates cardioprotective therapy

**Fig. 2:**
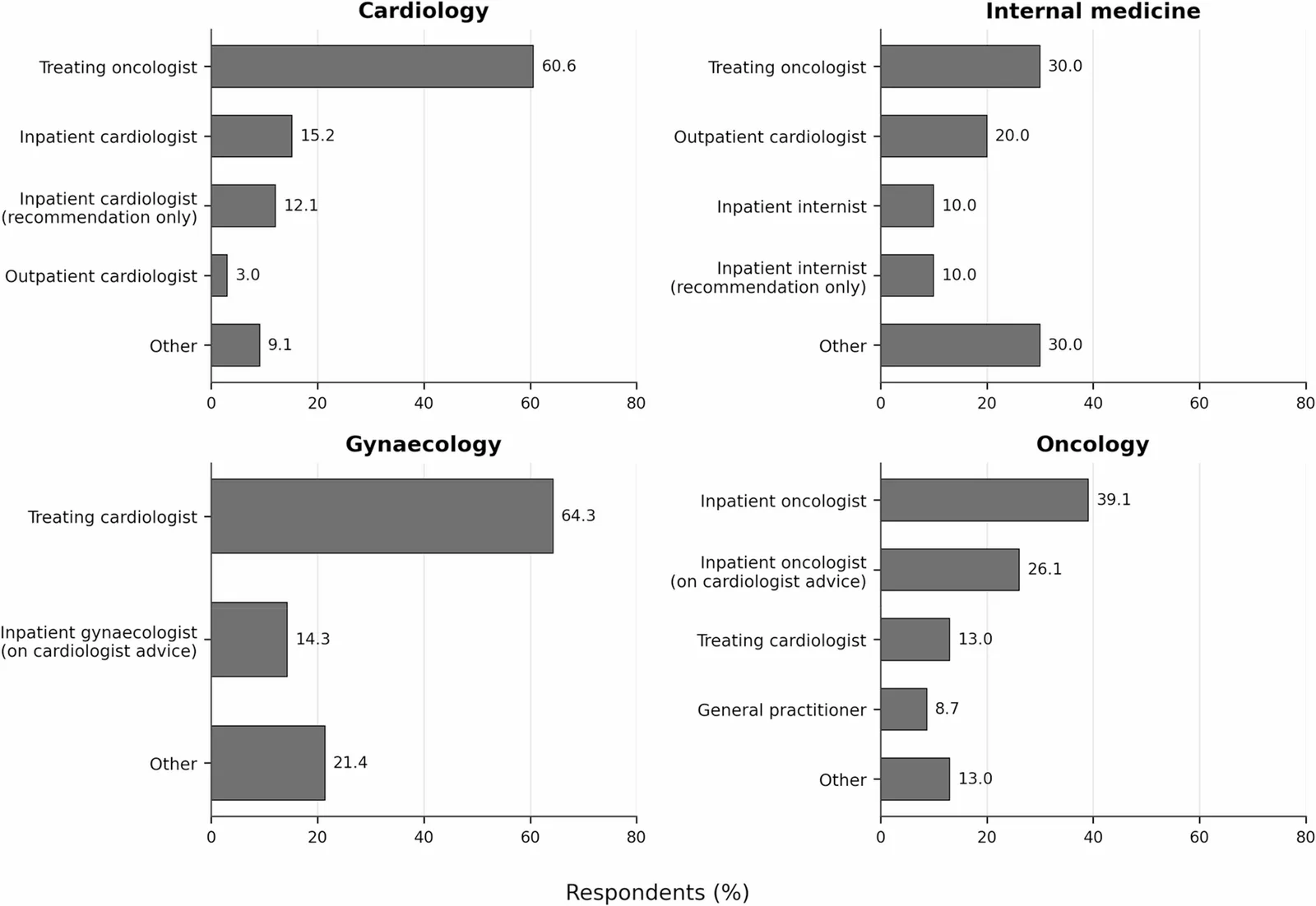
Reported initiator of cardioprotective therapy, by specialty. Cardiology *n* = 33, Internal medicine *n* = 10, Gynaecology *n* = 14, Oncology *n* = 23

The reported initiator of cardioprotective treatment is described within each specialty separately, because the response options were not identical across specialties (Fig. 2). Most cardiologists reported that initiation lay with the treating oncologist (60.6%). Among oncologists, most reported that the oncologist initiates therapy—either independently (39.1%) or acting on a cardiologist’s recommendation (26.1%), together about two-thirds. Most gynaecologists located initiation with the treating cardiologist (64.3%), whereas internists gave the most dispersed pattern. Because the specialties were offered different options, these accounts are not directly comparable, and the difference involving gynaecology is a difference between non-identical questions rather than a demonstrated mismatch in perceived responsibility. The item captured only who “initiates” and did not separate the distinct roles of identifying the indication, recommending an agent, prescribing or administering it, monitoring for adverse effects, and long-term titration; it therefore does not establish an allocation of responsibility.

### Interdisciplinary dialogue on cardiac findings

Cardiologists and internists were asked how they communicated cardiac-diagnostic findings and their therapeutic implications to treating oncologists (Additional file 3: Fig. S2). A consistently proactive, bidirectional dialogue—including by telephone and with a stated influence on therapy—was reported by only about one third of both groups (cardiology 33.3%, internal medicine 36.4%). Nearly half of cardiologists (48.5%) reserved such active dialogue for high-risk patients only, while a minority relied on written report letters without direct exchange (internal medicine 18.2%, cardiology 6.1%).

### Reasons for dissatisfaction with cardio-oncology care

Among gynaecologists and oncologists dissatisfied with the cardio-oncological care available to their patients, the cited reasons differed by specialty (Additional file 3: Fig. S3). Oncologists most often cited a lack of close monitoring (29.2%) and too few cardiology consultations (25.0%), whereas gynaecologists most often cited little dialogue or feedback for high-risk patients (27.8%) and the absence of clear statements on the cardiotoxic risk (22.2%). Across both groups the recurring themes were insufficient longitudinal monitoring and inadequate, non-specific communication.

## Discussion

This analysis complements our companion survey [12] by characterising the cardiological side of the cardio-oncology pathway: the points at which cardiology and internal medicine engage with it, the diagnostic thresholds cardiologists and internists apply, their use of strain imaging and biomarker surveillance, and their knowledge and use of cardioprotective pharmacotherapy. Five findings stand out. First, baseline risk stratification—the entry to the guideline pathway—was the step with the lowest cardiological involvement and the least clearly assigned ownership. Second, operational LVEF thresholds for flagging relevant cardiotoxicity were heterogeneous within each questionnaire version, and routine use of myocardial strain imaging (GLS) was not universal. Third, even where cardiology is engaged, structured surveillance in the standardised high-risk scenario—particularly in the late post-treatment window—is inconsistent. Fourth, heterogeneity and incomplete implementation were not confined to the treating specialties: within cardiology itself, self-reported thresholds for relevant cardiotoxicity were heterogeneous, structured surveillance late after treatment was reported by a minority, and acute symptoms remained a common trigger for biomarker testing. Fifth, the cardiac work-up that the treating specialties requested from cardiology was weighted towards imaging and ECG, whereas cardiac biomarkers and additional laboratory parameters that inform baseline risk classification were requested by a minority.

### Cardiological involvement and ownership of risk stratification

The 2022 ESC Guidelines state that baseline cardiovascular toxicity risk assessment should be considered by the treating oncology or haematology team for all patients scheduled to receive a cancer therapy with a clinically significant level of toxicity, or by a cardiologist where appropriate, with cardiological involvement thereafter determined by the resulting risk category [5]. Cardiological participation in the assessment of every patient is therefore not a guideline requirement, and our finding that formal risk stratification showed the lowest reported cardiological involvement of any step in the pathway (42.9%) should not be read as a departure from it. The mirror image is seen from the treating side, where 85.4% of gynaecologists and oncologists in the same survey reported performing risk stratification before chemotherapy [12]: the assessment is largely being made, but largely without cardiology. Viewed from the cardiological side, ownership was no clearer. Only about half of cardiologists and internists performed such stratification themselves (41.2% within an interdisciplinary team, 9.8% independently), 29.4% did not perform it at all, and 19.6% regarded it as generally the task of the treating oncologist or haematologist—a position consistent with the guideline pathway. What our data show is that consultants do not agree on where the task sits.

The assessment is nonetheless not wholly separable from cardiology. The HFA-ICOS proforma weights left ventricular function among the baseline risk factors, assigning high risk to an LVEF below 50% and moderate risk to an LVEF of 50–54% [4], so a complete classification requires access to a reliable assessment of left-ventricular function, which is often provided through cardiology or internal-medicine services. The question our findings raise is therefore not whether cardiology should see every patient, but whether the cardiac information required for classification reliably reaches the assessment, and whether the referral trigger that follows from it functions as intended. Our data speak to the first of these — the work-up requested from cardiology was weighted towards imaging and away from the biochemical components of risk assessment (Table 1) — and cannot address the second. Where an assessment was made, however, the interdisciplinary process worked much as intended: 88.5% of respondents reported that it was translated into a joint evaluation with the treating oncologist or gynaecologist, and only 7.7% issued a unilateral recommendation. What our data describe is therefore heterogeneity at the interface between the baseline assessment, the acquisition of the cardiac information it requires and the risk-based referral that should follow, rather than a deficit in the quality of the exchange once it exists; this favours structural remedies—such as referral criteria that trigger a formal HFA-ICOS-based assessment for defined regimens and risk profiles [4]—over general appeals to collaborate more closely [16].

In the work-up requested from cardiology by the treating specialties (Table 1), echocardiography and ECG predominated, whereas troponin, natriuretic peptides and additional laboratory parameters were requested by a minority, and roughly a quarter of respondents did not request an ECG. This matters because a baseline ECG is recommended in all patients before potentially cardiotoxic cancer therapy [5], and baseline cardiac biomarkers and cardiovascular risk factors such as diabetes, renal dysfunction and dyslipidaemia contribute to the HFA-ICOS risk category and guide subsequent management [4, 13]. The request to cardiology thus appears to be framed as an imaging question rather than as part of a complete baseline risk assessment, although the treating specialties may obtain some of these parameters themselves.

### Diagnostic thresholds and myocardial strain imaging

Our item did not reproduce the guideline definition of CTR-CVT, which is graded by severity, specifies a new LVEF reduction of at least 10% points to an LVEF of 40–49% for moderate dysfunction and a reduction to below 40% for severe dysfunction, and incorporates global longitudinal strain and cardiac biomarkers alongside LVEF [5]. It asked instead for the single echocardiographic threshold at which respondents assume relevant cardiotoxicity and communicate it, and it can therefore describe reporting thresholds in consulting practice but cannot measure concordance with the guideline.

Two observations survive that limitation. Within the version answered by cardiologists and internists — a single, identically worded item — responses were distributed across all five options, so consultants working from the same guideline and reporting to the same referring specialties did not share an operational threshold at which cardiotoxicity is flagged and communicated. And in both questionnaire versions a substantial proportion selected the option that any LVEF decline constitutes cardiotoxicity, a self-reported response rule that does not match the full ESC definition, since a small decline within the normal range does not by itself constitute CTR-CVT. Among cardiologists this response was given by 38.2%, among internists by 61.5%, among gynaecologists by 26.7% and among oncologists by 39.1%.

This matters beyond nomenclature. The threshold at which a cardiologist reports relevant cardiotoxicity is the threshold at which surveillance is intensified, cardioprotective treatment considered and anticancer therapy potentially modified. A criterion set below every guideline threshold may result in unnecessary interruption of effective oncological treatment; one set above them may delay recognition of dysfunction that the guidelines regard as actionable [7]. In our data both types of response rule were reported: among cardiologists, responses not matching the full ESC definition ran predominantly towards the more sensitive criterion, while a small group reported thresholds above every defined limit. That self-reported response rules not matching the full ESC definition were this frequent within the consulting specialties themselves—three years after the publication of a comprehensive guideline and in a predominantly academic sample—identifies education within cardiology as a target in its own right rather than one directed only at the treating specialties. Explicit, agreed LVEF and GLS reporting conventions embedded in referral criteria would reduce this variability.

### Biomarker surveillance in the standardised high-risk scenario

Serial troponin and natriuretic peptides are inexpensive, guideline-recommended tools that can detect cardiotoxicity before imaging changes and predict left-ventricular dysfunction [4, 9, 13]. In the high-risk scenario studied here, cardiologists reported baseline testing reasonably often, but late surveillance at 6–12 months—an important part of the first post-treatment year, during which much anthracycline-associated cardiotoxicity becomes clinically apparent [8]—was endorsed by only a minority even within cardiology in this predominantly academic sample, and acute symptoms remained a leading trigger. That a reactive pattern persists on the consulting side, and not only among treating oncologists, suggests that the problem is systemic: the pathway lacks a defined owner and schedule for post-treatment cardiovascular surveillance. Survivorship care plans with explicit biomarker and imaging intervals and clear handover between hospital-based and ambulatory providers are a concrete remedy.

### Cardioprotective pharmacotherapy

The evidence base for cardioprotection has grown from the early demonstration that troponin-guided enalapril prevents left-ventricular dysfunction [7], through neurohormonal prophylaxis in the PRADA trial [14], to statin protection during anthracycline therapy in patients with lymphoma in STOP-CA [15]; these trials are encouraging but remain context-specific rather than establishing broadly applicable primary cardioprotection across cancers and regimens. Against this background, the agents our respondents reported using were overwhelmingly generic heart-failure medications—ACE inhibitors, beta-blockers, MRAs and ARNI—familiar to both cardiology and oncology. In gynaecology, direct use of these agents was low despite many respondents reporting awareness of them, and most located responsibility for initiation with cardiology. Because the response options and the wording of the initiator item differed between specialties, and the item did not distinguish prescribing from recommendation or delegation, we describe these accounts within each specialty only and draw no comparison between them; the findings remain descriptive, do not permit conclusions about the adequacy of cardioprotective treatment and cannot distinguish delegation from non-provision, so non-use by a gynaecologist is not evidence of inadequate care where prescribing is delegated to cardiology. Explicitly assigning responsibility for prescribing and titrating neurohormonal cardioprotection, and naming it in shared referral pathways, would remove this ambiguity.

### Communication, perceived deficits and clinical implications

Consistently proactive, therapy-relevant interdisciplinary dialogue was reported by only about one third of cardiologists and internists, although a further 48.5% of cardiologists reported such dialogue for high-risk patients. Effective communication is repeatedly identified as a cornerstone of functioning cardio-oncology services [16, 17], because it is the mechanism by which divergent diagnostic thresholds are reconciled and treatment responsibilities are agreed at the point of care. Where that dialogue is only intermittent, as here, the threshold and responsibility gaps described above are unlikely to be corrected, and the same echocardiographic or biomarker finding may be acted upon differently by different specialties. The reasons gynaecologists and oncologists gave for dissatisfaction—insufficient monitoring, too few consultations and the absence of clear statements on cardiotoxic risk—map directly onto this communication deficit. Structured cardio-oncology services, with defined referral criteria and reporting standards, have been advocated precisely to address such fragmentation [16]. Building on existing guideline recommendations [5], pragmatic and largely resource-neutral steps include agreeing explicit LVEF and GLS reporting conventions, scheduling post-treatment biomarker and imaging surveillance within survivorship pathways, and naming a responsible discipline for prescribing and titrating cardioprotective therapy. Embedding a cardiologist in the existing oncological tumour board for patients with relevant cardiovascular risk offers a low-threshold way to convert episodic, reactive contact into structured dialogue [17]; whether such measures improve patient outcomes would ultimately need prospective evaluation.

### Strengths and limitations

The strengths of this analysis include its multicentre, multispecialty design, the use of a standardised clinical scenario, the reporting of exact counts and denominators, and full transparency about its relationship to the companion reports.

Several limitations must temper interpretation, of which the size and composition of the sample is the most important. All analyses are therefore exploratory and hypothesis-generating, and none is intended to support inference about German hospital practice as a whole. Confirmation would require a substantially larger sample, stratified by specialty and drawn so as to be regionally representative and to include community and basic-care hospitals in proportion to their share of oncological care. This is the more pertinent because academic teaching and university hospitals were over-represented among respondents, and the patterns described may not generalise to community and basic-care settings. Because access to cardio-oncology expertise and structured pathways is likely to be greater at academic centres, the gaps described here may be larger in non-academic and smaller hospitals; our data cannot test this.

The response rate of 8.4% is low in absolute terms, although rates of this order are usual for voluntary, unincentivised surveys of physicians: the survey of cardiology care delivered to cancer patients conducted by the ESC Council of Cardio-Oncology together with the Council for Cardiology Practice obtained 592 responses from approximately 45,000 cardiologists approached, a response rate of 1.3% [10]. Two features of our sampling frame mean that 8.4% underestimates the response rate among physicians who were both eligible and reached: successful delivery of invitations could not be verified owing to institutional spam filtering, and eligibility was defined at the level of specialty rather than of the individual department, so an unknown proportion of invitations will have reached departments to which the questionnaire did not apply. Neither consideration removes the risk of non-response bias, whose direction cannot be determined from our data.

The setting requires precise description. Respondents were hospital-employed physicians, but the questionnaire did not restrict answers to care delivered during an inpatient episode, and respondents were asked to estimate the proportion of their patients managed as outpatients. These findings therefore describe the practice of hospital-based physicians, a proportion of whose clinical work is ambulatory, and not inpatient care as such. The companion analysis [12] derives from the same survey; its characterisation of the setting as inpatient denotes the hospital-based employment of the respondents rather than a restriction of the reported practice to inpatient episodes.

Respondent characteristics beyond specialty and hospital category were not recorded: age, sex, seniority and years in post were not asked, in order to preserve anonymity in a survey addressed to one named physician per department. Age and career stage may plausibly influence familiarity with a guideline published in 2022, and we cannot examine this.

The measures reflect physicians’ accounts of their practice and were not verified against records; no patient-level care or outcomes were assessed, and social-desirability bias is possible. Investigations were reported as requested from cardiology rather than verified as performed. The instrument imposes further constraints: the item on “cardioprotective substances” combined prophylaxis, treatment of established disease and conventional cardiovascular indications across a heterogeneous drug list, and its wording did not distinguish cardioprotection in the narrow sense of preventing cancer therapy-related cardiac dysfunction from the broader prevention of cardiovascular complications of cancer therapy, under which antiplatelet agents and anticoagulants were offered; between-specialty comparisons of that item are descriptive only. The item on echocardiographic thresholds was administered in two non-equivalent versions and did not specify whether the LVEF decline was absolute or relative, so responses may not be interchangeable even within one version. The risk-factor item presented a selection from the HFA-ICOS proforma rather than the full instrument. Several items rest on small subgroups and are interpreted descriptively. The survey did not record the use of validated tools (e.g. HFA-ICOS) or patient sex, and, because much oncological care in Germany is ambulatory, these hospital-physician findings describe only part of the care pathway and should be read alongside the outpatient companion study [11]. Finally, this analysis and the companion report [12] draw on the same survey; the two analyses were pre-specified to address distinct questions using non-overlapping items (Additional file 2) and no result is duplicated, but conceptual proximity cannot be excluded and the papers should be read together.

## Conclusions

Respondents to this survey worked predominantly at academic teaching or university hospitals (73.3%), where access to cardio-oncology expertise is likely to be greatest; the gaps described below may therefore be larger in non-academic and smaller hospitals, which our data cannot test. In this cross-sectional survey of German hospital physicians, baseline risk stratification had the least clearly assigned ownership of any step in the pathway, although the guidelines assign the baseline assessment to the treating cancer specialty, diagnostic thresholds varied widely within each questionnaire version, with a substantial proportion of respondents in every specialty selecting a self-reported response rule that does not match the full ESC definition, routine use of strain imaging differed between cardiology and internal medicine, and late post-treatment biomarker testing in the standardised high-risk scenario was infrequent even among cardiologists. Because the survey captured physicians’ accounts of their practice rather than measured care or outcomes, these findings are exploratory and hypothesis-generating. They point to candidate targets for future evaluation—agreed LVEF and GLS reporting conventions, implementation of routine strain imaging, and structured post-treatment surveillance—whose effect on cancer therapy-related cardiovascular outcomes would need to be tested prospectively.

## Supplementary Information

Additional file 1. Full survey questionnaire — German original and English translation.

Additional file 2. Mapping of every survey item to its analysis and to the companion publications, identifying primary and secondary analyses.

Additional file 3. Supplementary material — Supplementary Figures S1 (involvement of cardiology and internal medicine across the treatment pathway), S2 (communication of cardiac findings to treating oncologists), S3 (reasons for dissatisfaction with cardio-oncological care) and S4 (familiarity with and use of cardiovascular drugs, by specialty); and Supplementary Tables S1 (reasons for not performing strain imaging), S2 (item-level denominators and missing data) and S3 (cardiovascular “cardioprotective” drugs, by specialty and indication). Table S2 reports, for every table and figure in the manuscript and the supplement, the number of respondents eligible to receive the item, the number who answered it, the number excluded by skip logic and the number lost to item non-response.

## Data Availability

No datasets were generated or analysed during the current study.

## Abbreviations

ACE: Angiotensin-converting enzyme
ARNI: Angiotensin receptor–neprilysin inhibitor
BNP: B-type natriuretic peptide
CTR-CVT: Cancer therapy-related cardiovascular toxicity
ECG: Electrocardiography
ESC: European Society of Cardiology
GFR: Glomerular filtration rate
GLS: Global longitudinal strain
HFA-ICOS: Heart Failure Association–International Cardio-Oncology Society
IQR: Interquartile range
LVEF: Left-ventricular ejection fraction
MRA: Mineralocorticoid-receptor antagonist
NT-proBNP: N-terminal pro-B-type natriuretic peptide
