# Supplementary material for: Cardio-oncology from the consulting side: diagnostic thresholds, surveillance and treatment responsibility among cardiologists and internists in a German multicentre survey
Source: Cardiooncology. 2026 Oct 7;12:123. doi: 10.1186/s40959-026-00588-7 (PMC13647822; doi:10.1186/s40959-026-00588-7)
Supplement: Supplementary file 1 [file 40959_2026_588_MOESM1_ESM.pdf]

## Additional file 1. Survey questionnaire

Supplementary material to: Cardio-oncology from the consulting side: diagnostic thresholds, surveillance and treatment responsibility among cardiologists and internists in a German multicentre survey.

Poley S, Payo-Anez L, Heidrich FM, et al.

*The questionnaire was administered online in German; the original instrument is reproduced below. Specialty-specific skip logic was applied, so that each respondent completed only the section corresponding to their department (Gynaecology, Internal Medicine, Cardiology or Oncology). Items were presented as single- or multiple-choice questions, Likert-scale ratings or free-text numeric entries, as indicated.*

### Fragebogen stationäre Kardio-Onkologie

In was für einer Abteilung sind Sie tätig?

*Bitte wählen Sie eine Antwort aus.*

- Gynäkologie
- Innere Medizin
- Kardiologie
- Onkologie

***Gynäkologie***

<u>Gynäkologie – 1:</u>

Wo sind Sie tätig?

*Bitte wählen Sie eine Antwort aus.*

- Universitätsklinik
- Universitätsklinik mit dezidierter kardioonkologischer Abteilung
- akademisches Lehrkrankenhaus
- Maximalversorger
- Grund- oder Regelversorger

<u>Gynäkologie – 2:</u>

Wie viele Patienten behandeln Sie ca. monatlich mit Krebs und kardiotoxischer Chemotherapie?

*Bitte geben Sie einen Zahlenwert an.*

- [offene Eingabe]

<u>Gynäkologie – 3:</u>

Wie viel Prozent dieser Patienten entfallen auf die unten aufgeführten Tumorentitäten?

*Bitte geben Sie schätzungsweise einen Wert für den Anteil der genannten Erkrankungen an der Gesamtzahl Ihrer Patienten mit Krebs an.*

- Brustkrebs [Prozentwert zwischen 0 bis 100]
- Gebärmutterkörperkrebs [Prozentwert zwischen 0 bis 100]
- Krebserkrankung der Ovarien und Adnexe [Prozentwert zwischen 0 bis 100]
- sonstige Krebserkrankungen [Prozentwert zwischen 0 bis 100]

<u>Gynäkologie – 4:</u>

Wie viel Prozent sind ambulante Patienten?

*Bitte geben Sie schätzungsweise einen Wert an.*

- Anteil ambulanter Patienten [Prozentwert zwischen 0 bis 100]

<u>Gynäkologie – 5:</u>

Welcher Anteil Ihrer Krebspatienten, die eine kardiotoxische Chemotherapie bekommen (Anthrazykline, HER2-Rezeptor-Antagonisten etc.), ist in kardiologischer Mitbetreuung?

*Bitte geben Sie einen Prozentwert an.*

- [offene Eingabe]

<u>Gynäkologie – 6:</u>

Was sind mögliche Gründe, warum nicht alle Patienten kardiologisch mitbetreut werden?

*Bitte wählen Sie die zutreffende(n) Antwort(en) aus.*

- keine Indikation zur kardiologischen Mitbetreuung
- fehlende kardiologische Kapazitäten
- fehlende therapeutische Konsequenzen
- Der Dialog mit den Kardiologen ist unzureichend, um daraus therapeutische Konsequenzen abzuleiten.
- sonstiges [offene Eingabe]

<u>Gynäkologie – 7:</u>

Wie erfolgt die kardiologische Mitbetreuung der Patienten?

*Bitte geben Sie schätzungsweise ein Verhältnis an.*

*Die Mitte der Skala entspricht einem Verhältnis von 50% ambulanter und 50% stationärer Mitbetreuung.*

- Verhältnis ambulanter zu stationärer Mitbetreuung [Prozentwert zwischen 0 bis 100]

Gynäkologie – 8:

In welchen Schritten der Behandlung von Patienten mit potentiell kardiotoxischer Chemotherapie involvieren Sie Kardiologen?

*Bitte wählen Sie die zutreffende(n) Antwort(en) aus.*

- kardiotoxische Risikostratifizierung der Chemotherapie
- kardiologische Diagnostik vor der Chemotherapie
- kardiologische Diagnostik während der Chemotherapie
- kardiologische Diagnostik nach der Chemotherapie
- Anpassung der Therapie bei eingetretener Kardiotoxizität
- sonstiges [offene Eingabe]

Gynäkologie – 9:

Führen Sie vor dem Beginn einer Chemotherapie eine Risikostratifizierung des kardiotoxischen Risikos durch?

*Bitte wählen Sie eine Antwort aus.*

- ja
- nein

Gynäkologie – 10:

Welche Parameter fließen in die Risikobewertung des kardiotoxischen Risikos mit ein?

*Bitte wählen Sie die zutreffende(n) Antwort(en) aus.*

- kardiale Vorerkrankungen (z.B. Herzinsuffizienz, KHK)
- Alter (>60 Jahre)
- Geschlecht
- Diabetes mellitus
- Niereninsuffizienz
- Übergewicht
- hohe Dosis der Anthrazyklin Chemotherapie
- zusätzliche Radiotherapie, wobei das Herz im Bestrahlungsfeld ist

- HER2-Inhibitoren, Proteasom-Inhibitoren, VEGF-Therapie
- erhöhte kardiale Biomarker
- linksventrikuläre Pumpfunktion
- sonstige [offene Eingabe]

Gynäkologie – 11:

Im Falle dass eine Risikoerhebung des kardiotoxischen Risikos durchgeführt wird, hat dies einen Einfluss auf die Auswahl der Chemotherapie?

*Bitte wählen Sie eine Antwort aus.*

- nein
- ja, ich passe allein die Chemotherapie an
- ja, ggf. konsultiere ich einen Kardiologen und das kardiotoxische Risiko wird für jeden Patienten gemeinsam evaluiert und die Therapie besprochen

Gynäkologie – 12:

Was sind Gründe dafür, dass keine Risikostratifizierung von Ihnen durchgeführt wird?

[Mehrfachauswahl möglich]

- Das ist die Aufgabe des betreuenden Kardiologen.
- Es fehlt an Zeit diese Erhebung durchzuführen.
- Es fehlt an den nötigen Instrumenten diese Erhebung durchzuführen.
- Die Interpretation der Ergebnisse ist zu komplex.
- Die Erhebung ist zu kostenintensiv.
- sonstiges [offene Eingabe]

Gynäkologie – 13:

Welche Untersuchungen werden auf Ihre Veranlassung vom Kardiologen durchgeführt?

*Bitte wählen Sie die zutreffende(n) Antwort(en) aus.*

- Anamnese
- EKG
- Echokardiographie
- Ergometrie

- Labor TNT
- Labor BNP
- Laborparameter wie z.B. Blutglukose, GFR, Lipidprofil
- sonstiges [offene Eingabe]

Gynäkologie – 14:

Gehen Sie von einer Patientin aus, die eine Anthrazyklin Chemotherapie bekommt. Eine Herzinsuffizienz ist bereits bekannt. Wie oft wird diese Patientin zur kardiologischen Konsultation vorgestellt?

*Bitte wählen Sie die zutreffende(n) Antwort(en) aus.*

- vor Einleitung der Chemotherapie
- nach jedem Zyklus der Therapie
- nach jedem 2. Zyklus der Therapie
- nach Abschluss der Chemotherapie
- 3 Monate nach Abschluss der Therapie
- 6 Monate nach Abschluss der Therapie
- 12 Monate nach Abschluss der Therapie
- 24 Monate nach Abschluss der Therapie
- bei akuten kardialen Beschwerden
- sonstiges [offene Eingabe]

Gynäkologie – 15:

Gibt es zwischen Ihnen und den betreuenden Kardiologen einen engen Dialog über die Ergebnisse der kardialen Diagnostik und die Implikationen auf die daraus resultierende Therapie (sowohl kardiologisch und onkologisch)?

*Bitte wählen Sie eine Antwort aus.*

- ja, es besteht immer ein aktiver Dialog ggf. auch telefonisch, welcher auch Einfluss auf die Therapie hat
- ja, es besteht ein aktiver Dialog ggf. auch telefonisch bei bestimmen Risikopatienten, welcher auch Einfluss auf die Therapie hat
- nein, ich bekomme dezidierte therapeutische Ratschläge nur im Befundbrief

- nein, ich muss alle wesentlichen Informationen aus der durchgeführten Diagnostik selbst interpretieren
- sonstiges [offene Eingabe]

Gynäkologie – 16:

Beziehen Sie bei Patienten mit vermuteter chemotherapiebedingter Kardiotoxizität Kardiologen in die weitere Abwägung der Fortführung/Anpassung der Chemotherapie mit ein?

*Bitte wählen Sie eine Antwort aus.*

- immer
- gelegentlich
- ja, aber nur in Ausnahmefällen
- in der Regel nicht

Gynäkologie – 17:

Bei welchen echokardiographischen Kriterien gehen Sie von einer relevanten Kardiotoxizität aus?

*Bitte wählen eine Antwort aus.*

- LV-EF Abfall > 40%
- LV-EF Abfall > 30%
- LV-EF Abfall > 20%
- LV-EF Abfall > 10%
- jeder LV-EF Abfall stellt eine Kardiotoxizität dar

Gynäkologie – 18:

Bestimmen Sie NT-ProBNP oder Troponin und wenn ja, bei wie viel Prozent der behandelten *Patienten vor einer Chemotherapie haben Sie selbst NT-ProBNP oder Troponin bestimmt?*

*Bitte wählen Sie die zutreffende(n) Antwort(en) aus.*

*Bitte geben Sie ggf. den Anteil an behandelten Patienten (in Prozent) an, bei welchem Sie selbst vor einer Chemotherapie NT-ProBNP oder Troponin bestimmt haben.*

- nein, es wird kein NT-ProBNP oder Troponin bestimmt
- ja, es wird NT-ProBNP bestimmt [offene Eingabe]
- ja, es wird Troponin bestimmt [offene Eingabe]

Gynäkologie – 19:

Was sind die Gründe, warum diese Werte nicht bestimmt werden?

*Bitte wählen Sie die zutreffende(n) Antwort(en) aus.*

- zu hohe Kosten
- wenig Expertise bei der Interpretation der Ergebnisse
- das sollte Aufgabe des Kardiologen sein
- sonstiges [offene Eingabe]

Gynäkologie – 20:

Gehen Sie wieder von einer Patientin aus, die eine Anthrazyklin Chemotherapie bekommt. Eine Herzinsuffizienz ist bereits bekannt. Wie häufig bestimmen Sie diese Herzwerte?

*Bitte wählen Sie die zutreffende(n) Antwort(en) aus.*

- vor Einleitung der Chemotherapie
- nach jedem Zyklus der Therapie
- nach jedem 2. Zyklus der Therapie
- nach Abschluss der Chemotherapie
- 3 Monate nach Abschluss der Therapie
- 6 Monate nach Abschluss der Therapie
- 12 Monate nach Abschluss der Therapie
- 24 Monate nach Abschluss der Therapie
- bei akuten kardialen Beschwerden
- sonstiges [offene Eingabe]

Gynäkologie – 21:

Kennen und verwenden Sie kardioprotektive Substanzen in der Behandlung onkologischer Patienten?

*Bitte wählen Sie eine Antwort aus.*

- ja, ich kenne und verwende kardioprotektive Substanzen in der Behandlung onkologischer Patienten
- ich kenne kardioprotektive Substanzen in der Behandlung onkologischer Patienten, aber verwende keine kardioprotektiven
- Substanzen in der Behandlung onkologischer Patienten

- nein, ich kenne und verwende keine kardioprotektiven Substanzen in der Behandlung onkologischer Patienten

<u>Gynäkologie – 22:</u>

Welche kardioprotektiven Substanzen kennen und verwenden Sie bei Ihren Patienten?

*Bitte wählen Sie die zutreffende(n) Antwort(en) aus.*

- ACE-Hemmer (z.B. Enalapril)
- Beta-Blocker (z.B. Carvedilol)
- Aldosteron Antagonisten (z.B. Spironolacton)
- Statine (z.B. Simvastatin)
- Eisenchelatoren (z.B. Dexrazoxane)
- Thromboyztenaggregationshemmer (z.B. Aspirin)
- Antikoagulantien (z.B. Enoxaparin, Rivaroxaban)
- Neprilysin Inhibitoren
- sonstige [offene Eingabe]

<u>Gynäkologie – 23:</u>

Wer initiiert die kardioprotektive Behandlung in der Regel bei diesen Patienten?

- *Bitte wählen Sie eine Antwort aus.*
- der betreuende Kardiologe
- der Hausarzt
- ich selbst
- ich selbst auf Empfehlung des Kardiologen
- sonstige [offene Eingabe]

<u>Gynäkologie – 24:</u>

Wie zufrieden sind Sie mit der kardiologischen Betreuung Ihrer Patienten?

*Bitte wählen Sie eine Antwort aus.*

*1 – gering*

*10 – sehr zufrieden*

- [Skala von 1 bis 10]

Gynäkologie – 25:

Was sind Gründe, warum Sie unzufrieden sind?

*Bitte wählen Sie die zutreffende(n) Antwort(en) aus.*

*Überspringen Sie bitte diese Frage, wenn Sie bei der Frage zuvor 9 oder 10 (sehr zufrieden) ausgewählt haben*.

- fehlende engmaschige Betreuung
- zu wenig kardiologische Konsultationen
- wenig Dialog/Rückmeldung bei Hochrisikopatienten
- keine klaren Aussagen/Festlegungen zum kardiotoxischen Risiko
- sonstiges [offene Eingabe]

Gynäkologie – 26:

Analog zu verschiedenen Boards in einer Klinik, gibt es in Ihrer Klinik ein regelmäßiges kardioonkologisches Board?

Wenn ja, wie häufig findet dieses statt?

*Bitte wählen Sie eine Antwort aus*

- nein
- ja, täglich
- ja, 1 Mal pro Woche
- ja, 2 Mal pro Monat
- ja, monatlich
- ja, 1 Mal pro Quartal
- sonstiges [offene Eingabe]

Gynäkologie – 27:

Halten Sie kardioonkologische Boards für sinnvoll?

*Bitte wählen Sie eine Antwort aus.*

- ja, sehr sinnvoll für jeden Patienten
- ja, aber nur für Hochrisikopatienten
- nein, die therapeutische Konsequenz wäre zu gering

Gynäkologie – 28:

Wie kann die Betreuung kardioonkologischer Patienten aus Ihrer Sicht verbessert werden?

*Bitte wählen Sie die zutreffende(n) Antwort(en) aus.*

- Simplifizierung der Leitlinien
- digitale Tools zur Risikostratifizierung
- Tools zur besseren Bestimmung der Kardiotoxizität
- engere Kommunikation mit behandelnden Kardiologen
- regelmäßige interdisziplinäre Fortbildungen
- Kontaktmöglichkeit zu kardioonkologischem Expertennetzwerk bei schwierigen Fragestellungen
- Tools zur Überwachung des kardialen Status
- sonstiges

***Kardiologie***

Kardiologie – 1:

Wo sind Sie tätig?

*Bitte wählen Sie eine Antwort aus.*

- Universitätsklinik
- Universitätsklinik mit dezidierter kardioonkologischer Abteilung
- Akademisches Lehrkrankenhaus
- Maximalversorger
- Grund- oder Regelversorger

Kardiologie – 2:

Wie viele Patienten mit Chemotherapie werden Ihnen zur Diagnostik/kardiologischen Mitbetreuung durchschnittlich monatlich vorgestellt?

*Bitte geben Sie einen Zahlenwert an.*

- [offene Eingabe]

Kardiologie – 3:

Wie viel Prozent dieser Patienten entfallen auf die unten aufgeführten Tumorentitäten?

*Bitte geben Sie schätzungsweise einen Wert für den Anteil der genannten Erkrankungen an der Gesamtzahl Ihrer Patienten mit Krebs an.*

- Brustkrebs [Prozentwert zwischen 0 bis 100]
- Prostatakrebs [Prozentwert zwischen 0 bis 100]
- Darmkrebs [Prozentwert zwischen 0 bis 100]
- Lungenkrebs [Prozentwert zwischen 0 bis 100]
- Harnblasenkrebs [Prozentwert zwischen 0 bis 100]
- Nierenkrebs [Prozentwert zwischen 0 bis 100]
- Krebs von Mundhöhle und Rachen [Prozentwert zwischen 0 bis 100]
- Leukämien [Prozentwert zwischen 0 bis 100]
- Non-Hodgkin-Lymphome [Prozentwert zwischen 0 bis 100]
- sonstige Krebserkrankungen [Prozentwert zwischen 0 bis 100]

<u>Kardiologie – 4:</u>

Wie viel Prozent sind ambulante Patienten?

*Bitte geben Sie schätzungsweise einen Wert an.*

- Anteil ambulanter Patienten [Prozentwert zwischen 0 bis 100]

<u>Kardiologie – 5:</u>

In welchen Schritten der Behandlung von Patienten mit potentiell kardiotoxischer Chemotherapie sind Sie involviert?

*Bitte wählen Sie die zutreffende(n) Antwort(en) aus.*

- kardiotoxische Risikostratifizierung der Chemotherapie
- kardiologische Diagnostik vor der Chemotherapie
- kardiologische Diagnostik während der Chemotherapie
- kardiologische Diagnostik nach der Chemotherapie
- Initiierung der Therapie bei eingetretener Kardiotoxizität
- Anpassung der Therapie bei eingetretener Kardiotoxizität
- Tumorboards

<u>Kardiologie – 6:</u>

Führen Sie eine Stratifizierung des potentiell kardiotoxischen Risikos der Chemotherapie durch?

*Bitte wählen Sie eine Antwort aus.*

- ja, allein
- ja, aber in Zusammenarbeit mit einem interdisziplinären Team
- nein
- nein, das ist generell die Aufgabe des betreuenden fachspezifischen Onkologen/Hämatologen

<u>Kardiologie – 7:</u>

Hat die von Ihnen durchgeführte Risikoerhebung des kardiotoxischen Risikos einen Einfluss auf die Auswahl der Chemotherapie der Patienten?

*Bitte wählen Sie eine Antwort aus.*

- ja, ich gebe allein dezidierte Empfehlungen zur Anpassung der Therapie
- ja, ggf. konsultiere ich den Onkologen/Gynäkologen und das kardiotoxische Risiko wird für jeden Patienten gemeinsam evaluiert
- nein, es erfolgt keinerlei Kommunikation

<u>Kardiologie – 8:</u>

Welche Parameter fließen in die Risikobewertung des kardiotoxischen Risikos mit ein?

*Bitte wählen Sie die zutreffende(n) Antwort(en) aus.*

- kardiale Vorerkrankungen (z.B. Herzinsuffizienz, KHK)
- Alter (>60 Jahre)
- Geschlecht
- Diabetes mellitus
- Niereninsuffizienz
- Übergewicht
- hohe Dosis der Anthrazyklin Chemotherapie
- zusätzliche Radiotherapie, wobei das Herz im Bestrahlungsfeld ist
- HER2 Inhibitoren, Proteasom Inhibitoren, VEGF Therapie
- erhöhte kardiale Biomarker
- linksventrikuläre Pumpfunktion

- sonstige [offene Eingabe]

Kardiologie – 9:

Welche Untersuchungen führen Sie standardmäßig bei diesen Patienten durch?

*Bitte wählen Sie die zutreffende(n) Antwort(en) aus.*

- Anamnese
- EKG
- Echokardiographie
- Ergometrie
- Labor TNT
- Labor BNP
- Laborparameter wie z.B. Blutglukose, GFR, Lipidprofil
- sonstiges [offene Eingabe]

Kardiologie – 10:

Gehen Sie von einer Patientin aus, die eine Anthrazyklin Chemotherapie bekommt. Eine Herzinsuffizienz ist bereits bekannt.

Wie oft wird Ihnen diese Patientin vorgestellt?

*Bitte wählen Sie die zutreffende(n) Antwort(en) aus.*

- vor Einleitung der Chemotherapie
- nach jedem Zyklus der Therapie
- nach jedem 2. Zyklus der Therapie
- nach Abschluss der Chemotherapie
- 3 Monate nach Abschluss der Therapie
- 6 Monate nach Abschluss der Therapie
- 12 Monate nach Abschluss der Therapie
- 24 Monate nach Abschluss der Therapie
- bei akuten kardialen Beschwerden
- sonstiges [offene Eingabe]

Kardiologie – 11:

Werden Sie bei Patienten mit vermuteter chemotherapiebedingter Kardiotoxizität in die weitere Abwägung der Fortführung/Anpassung der Chemotherapie mit einbezogen?

*Bitte wählen Sie eine Antwort aus.*

- immer
- gelegentlich
- ja, aber nur in Ausnahmefällen
- nein

Kardiologie – 12:

Bei welchen echokardiographischen Kriterien gehen Sie von einer relevanten Kardiotoxizität aus und kommunizieren das?

*Bitte wählen Sie eine Antwort aus.*

- LV-EF Abfall > 40% zu einem Wert unterhalb der Grenzwerte
- LV-EF Abfall > 30% zu einem Wert unterhalb der Grenzwerte
- LV-EF Abfall > 20% zu einem Wert unterhalb der Grenzwerte
- LV-EF Abfall > 10% zu einem Wert unterhalb der Grenzwerte
- jeder LV-EF Abfall stellt eine Kardiotoxizität dar

Kardiologie – 13:

Nutzen Sie neben der LV-EF Bestimmung regelhaft echokardiographische Strainuntersuchungen zur kardialen Verlaufskontrolle nach Chemotherapie?

*Bitte wählen Sie eine Antwort aus.*

- ja
- nein

Kardiologie – 14:

Was sind Gründe, warum keine Strainuntersuchungen stattfinden?

*Bitte wählen Sie die zutreffende(n) Antwort(en) aus.*

- fehlende technische Ausrüstung
- zu zeitaufwendig
- Schallbedingungen oft nicht ausreichend
- therapeutische Konsequenz unklar

- fehlende Expertise
- fehlendes Personal
- sonstiges [offene Eingabe]

Kardiologie – 15:

Bestimmen Sie NT-ProBNP oder Troponin und wenn ja, bei wie viel Prozent der behandelten Patienten vor einer Chemotherapie haben Sie selbst NT-ProBNP oder Troponin bestimmt?

*Bitte wählen Sie die zutreffende(n) Antwort(en) aus.*

*Bitte geben Sie ggf. den Anteil an behandelten Patienten (in Prozent) an, bei welchem Sie selbst vor einer Chemotherapie NT-ProBNP oder Troponin bestimmt haben.*

- nein, es wird kein NT-ProBNP oder Troponin bestimmt
- ja, es wird NT-ProBNP bestimmt [offene Eingabe]
- ja, es wird Troponin bestimmt [offene Eingabe]

Kardiologie – 16:

Gehen Sie wieder von einer Patientin aus, die eine Anthrazyklin Chemotherapie bekommt. Eine Herzinsuffizienz ist bereits bekannt. Wie häufig bestimmen Sie diese Herzwerte?

*Bitte wählen Sie die zutreffende(n) Antwort(en) aus.*

- vor Einleitung der Chemotherapie
- nach jedem Zyklus der der Therapie
- nach jedem 2. Zyklus der Therapie
- nach Abschluss der Chemotherapie
- 3 Monate nach Abschluss der Therapie
- 6 Monate nach Abschluss der Therapie
- 12 Monate nach Abschluss der Therapie
- bei akuten kardialen Beschwerden
- bei: [offene Eingabe]
- nein, es werden keine Werte bestimmt

Kardiologie – 17:

Was sind die Gründe, warum diese Werte nicht bestimmt werden?

*Bitte wählen Sie die zutreffende(n) Antwort(en) aus.*

- Das sollte Aufgabe des Onkologen sein.
- zu hohe Kosten
- Die therapeutische Konsequenz ist unklar.
- wenig Expertise bei der Interpretation der Ergebnisse
- sonstiges [offene Eingabe]

<u>Kardiologie – 18:</u>

Kennen und verwenden Sie kardioprotektive Substanzen in der Behandlung onkologischer Patienten?

*Bitte wählen Sie eine Antwort aus.*

- ja, ich kenne und verwende kardioprotektive Substanzen in der Behandlung onkologischer Patienten
- ich kenne kardioprotektive Substanzen in der Behandlung onkologischer Patienten, aber verwende keine kardioprotektiven
- Substanzen in der Behandlung onkologischer Patienten
- nein, ich kenne und verwende keine kardioprotektiven Substanzen in der Behandlung onkologischer Patienten

<u>Kardiologie – 19:</u>

Welche Substanzen kennen und verwenden Sie bei Ihren Patienten?

*Bitte wählen Sie die zutreffende(n) Antwort(en) aus.*

- ACE-Hemmer (z.B. Enalapril)
- Beta-Blocker (z.B. Carvedilol)
- ARNI (z.B. Entresto)
- Aldosteron Antagonisten (z.B. Spironolacton)
- Statine (z.B. Atorvastatin)
- Eisenchelatoren (z.B. Dexrazoxan)
- Thrombozytenaggregationshemmer (z.B. Aspirin)
- Antikoagulantien (z.B. Enoxaparin, Rivaroxaban)
- Neprilysin Inhibitoren
- sonstige [offene Eingabe]

Kardiologie – 20:

Wer initiiert die kardioprotektive Behandlung in der Regel bei diesen Patienten?

*Bitte wählen Sie eine Antwort aus.*

- der betreuende Onkologe
- der ambulante Kardiologe
- der Hausarzt
- ich selbst
- ich formuliere lediglich eine Empfehlung
- sonstige [offene Eingabe]

Kardiologie – 21:

Gibt es zwischen Ihnen und den betreuenden Onkologen einen engen Dialog über die Ergebnisse der kardialen Diagnostik und die Implikationen auf die daraus resultierende Therapie (sowohl kardiologisch und onkologisch)?

*Bitte wählen Sie eine Antwort aus.*

- ja, es besteht immer ein aktiver Dialog ggf. auch telefonisch, welcher auch Einfluss auf die Therapie hat
- ja, es besteht ein aktiver Dialog ggf. auch telefonisch bei bestimmen Risikopatienten, welcher auch Einfluss auf die Therapie hat
- nein, ich gebe dezidierte therapeutische Ratschläge im Befundbrief
- nein, alle wesentlichen Informationen sollten die behandelnden Onkologen/Gynäkologen aus der durchgeführten Diagnostik
- selbst interpretieren
- sonstiges [offene Eingabe]

Kardiologie – 22:

Ist aus Ihrer Sicht eine spezialisierte kardioonkologische Betreuung von Patienten mit Chemotherapie sinnvoll?

*Bitte wählen Sie eine Antwort aus.*

*Bitte geben Sie ggf. eine Begründung an.*

- ja [offene Eingabe]
- nein [offene Eingabe]

Kardiologie – 23:

Analog zu verschiedenen Boards in einer Klinik, gibt es in Ihrer Klinik ein regelmäßiges kardioonkologisches Board?

Wenn ja wie häufig findet dieses statt?

*Bitte wählen Sie Antwort aus.*

*Bitte geben Sie ggf. die Häufigkeit an.*

- ja [offene Eingabe]
- nein

Kardiologie – 24:

Halten Sie kardioonkologische Boards für sinnvoll?

*Bitte wählen Sie ein Antwort aus.*

- ja, sehr sinnvoll für jeden Patienten
- ja, aber nur sinnvoll für Hochrisikopatienten
- nein, die therapeutische Konsequenz wäre zu gering

Kardiologie – 25:

Wie schätzen Sie die interdisziplinäre Betreuung kardioonkologischer Patienten bei Ihnen in der Klinik ein?

*Bitte geben Sie an wie viel Prozent bis zur optimalen Betreuung fehlen.*

- Prozent bis zur optimalen Betreuung [Prozentwert zwischen 0 bis 100]

Kardiologie – 26:

Wie kann die Betreuung kardioonkologischer Patienten aus Ihrer Sicht verbessert werden?

*Bitte wählen Sie die zutreffende(n) Antwort(enI aus.*

- Simplifizierung der Leitlinien
- digitale Tools zur Risikostratifizierung
- Tools zur besseren Bestimmung der Kardiotoxizität
- engere Kommunikation mit behandelnden Onkologen
- regelmäßige interdisziplinäre Fortbildungen
- Kontaktmöglichkeit zu kardioonkologischem Expertennetzwerk bei schwierigen Fragestellungen

- Tools zur Überwachung des kardialen Status
- sonstiges [offene Eingabe]

***Onkologie***

Onkologie – 1:

Wo sind Sie tätig?

*Bitte wählen Sie eine Antwort aus.*

- Universitätsklinik
- Universitätsklinik mit dezidierter kardioonkologischer Abteilung
- akademisches Lehrkrankenhaus
- Maximalversorger
- Grund- oder Regelversorger

Onkologie – 2:

Wie viele Patienten behandeln Sie ca. monatlich mit Krebs und kardiotoxischer Chemotherapie?

*Bitte geben Sie einen Zahlenwert an.*

- [offene Eingabe]

Onkologie – 3:

Wie viel Prozent dieser Patienten entfallen auf die unten aufgeführten Tumorentitäten?

*Bitte geben Sie schätzungsweise einen Wert für den Anteil der genannten Erkrankungen an der Gesamtzahl Ihrer Patienten mit Krebs an.*

- Prostatakrebs [Prozentwert zwischen 0 bis 100]
- Darmkrebs [Prozentwert zwischen 0 bis 100]
- Lungenkrebs [Prozentwert zwischen 0 bis 100]
- Harnblasenkrebs [Prozentwert zwischen 0 bis 100]
- Nierenkrebs [Prozentwert zwischen 0 bis 100]
- Krebs von Mundhöhle und Rachen [Prozentwert zwischen 0 bis 100]
- Leukämien [Prozentwert zwischen 0 bis 100]
- Non-Hodgkin-Lymphome [Prozentwert zwischen 0 bis 100]
- sonstige Krebserkrankungen [Prozentwert zwischen 0 bis 100]

Onkologie – 4:

Wie viel Prozent sind ambulante Patienten?

*Bitte geben Sie schätzungsweise einen Wert an.*

- Anteil ambulanter Patienten [Prozentwert zwischen 0 bis 100]

Onkologie – 5:

Welcher Anteil Ihrer Krebspatienten, die eine kardiotoxische Chemotherapie bekommen (Anthrazykline, HER2-Rezeptor-Antagonisten etc.), ist in kardiologischer Mitbetreuung?

*Bitte geben Sie einen Prozentwert an.*

- [offene Eingabe]

Onkologie – 6:

Was sind mögliche Gründe, warum nicht alle Patienten kardiologisch mitbetreut werden?

*Bitte wählen Sie die zutreffende(n) Antwort(en) aus.*

- keine Indikation zur kardiologischen Mitbetreuung
- fehlende kardiologische Kapazitäten
- fehlende therapeutische Konsequenzen
- Der Dialog mit den Kardiologen ist unzureichend, um daraus therapeutische Konsequenzen abzuleiten.
- sonstiges [offene Eingabe]

Onkologie – 7:

Wie erfolgt die kardiologische Mitbetreuung der Patienten?

*Bitte geben Sie schätzungsweise ein Verhältnis an.*

*Die Mitte der Skala entspricht einem Verhältnis von 50% ambulanter und 50% stationärer Mitbetreuung.*

- Verhältnis ambulanter zu stationärer Mitbetreuung [Prozentwert zwischen 0 bis 100]

Onkologie – 8:

In welchen Schritten der Behandlung von Patienten mit potentiell kardiotoxischer Chemotherapie involvieren Sie Kardiologen?

*Bitte wählen Sie die zutreffende(n) Antwort(en) aus.*

- kardiotoxische Risikostratifizierung der Chemotherapie
- kardiologische Diagnostik vor der Chemotherapie

- kardiologische Diagnostik während der Chemotherapie
- kardiologische Diagnostik nach der Chemotherapie
- Anpassung der Therapie bei eingetretener Kardiotoxizität
- sonstiges [offene Eingabe]

Onkologie – 9:

Führen Sie vor dem Beginn einer Chemotherapie eine Risikostratifizierung des kardiotoxischen Risikos durch?

*Bitte wählen Sie eine Antwort aus.*

- ja
- nein

Onkologie – 10:

Welche Parameter fließen in die Risikobewertung des kardiotoxischen Risikos mit ein?

*Bitte wählen Sie die zutreffende(n) Antwort(en) aus.*

- kardiale Vorerkrankungen (z.B. Herzinsuffizienz, KHK)
- Alter (>60 Jahre)
- Geschlecht
- Diabetes mellitus
- Niereninsuffizienz
- Übergewicht
- hohe Dosis der Anthrazyklin Chemotherapie
- zusätzliche Radiotherapie, wobei das Herz im Bestrahlungsfeld ist
- HER2-Inhibitoren, Proteasom-Inhibitoren, VEGF-Therapie
- erhöhte kardiale Biomarker
- linksventrikuläre Pumpfunktion
- sonstige [offene Eingabe]

Onkologie – 11:

Im Falle dass eine Risikoerhebung des kardiotoxischen Risikos durchgeführt wird, hat dies einen Einfluss auf die Auswahl der Chemotherapie?

*Bitte wählen Sie eine Antwort aus.*

- nein
- ja, ich passe allein die Chemotherapie an
- ja, ggf. konsultiere ich einen Kardiologen und das kardiotoxische Risiko wird für jeden Patienten gemeinsam evaluiert und die
- Therapie besprochen

<u>Onkologie – 12:</u>

Was sind Gründe dafür, dass keine Risikostratifizierung von Ihnen durchgeführt wird?

[Mehrfachauswahl möglich]

- Das ist die Aufgabe des betreuenden Kardiologen.
- Es fehlt an Zeit diese Erhebung durchzuführen.
- Es fehlt an den nötigen Instrumenten diese Erhebung durchzuführen.
- Die Interpretation der Ergebnisse ist zu komplex.
- Die Erhebung ist zu kostenintensiv.
- sonstiges [offene Eingabe]

<u>Onkologie – 13:</u>

*Welche Untersuchungen werden auf Ihre Veranlassung vom Kardiologen durchgeführt?*

*Bitte wählen Sie die zutreffende(n) Antwort(en) aus.*

- Anamnese
- EKG
- Echokardiographie
- Ergometrie
- Labor TNT
- Labor BNP
- Laborparameter wie z.B. Blutglukose, GFR, Lipidprofil
- sonstiges [offene Eingabe]

<u>Onkologie – 14:</u>

Gehen Sie von einer Patientin aus, die eine Anthrazyklin Chemotherapie bekommt. Eine Herzinsuffizienz ist bereits bekannt. Wie oft wird diese Patientin zur kardiologischen Konsultation vorgestellt?

*Bitte wählen Sie die zutreffende(n) Antwort(en) aus.*

- vor Einleitung der Chemotherapie
- nach jedem Zyklus der Therapie
- nach jedem 2. Zyklus der Therapie
- nach Abschluss der Chemotherapie
- 3 Monate nach Abschluss der Therapie
- 6 Monate nach Abschluss der Therapie
- 12 Monate nach Abschluss der Therapie
- 24 Monate nach Abschluss der Therapie
- bei akuten kardialen Beschwerden
- sonstiges [offene Eingabe]

<u>Onkologie – 15:</u>

Gibt es zwischen Ihnen und den betreuenden Kardiologen einen engen Dialog über die Ergebnisse der kardialen Diagnostik und die Implikationen auf die daraus resultierende Therapie (sowohl kardiologisch und onkologisch)?

*Bitte wählen Sie eine Antwort aus.*

- ja, es besteht immer ein aktiver Dialog ggf. auch telefonisch, welcher auch Einfluss auf die Therapie hat
- ja, es besteht ein aktiver Dialog ggf. auch telefonisch bei bestimmen Risikopatienten, welcher auch Einfluss auf die Therapie hat
- nein, ich bekomme dezidierte therapeutische Ratschläge nur im Befundbrief
- nein, ich muss alle wesentlichen Informationen aus der durchgeführten Diagnostik selbst interpretieren
- sonstiges [offene Eingabe]

<u>Onkologie – 16:</u>

Beziehen Sie bei Patienten mit vermuteter chemotherapiebedingter Kardiotoxizität Kardiologen in die weitere Abwägung der Fortführung/Anpassung der Chemotherapie mit ein?

*Bitte wählen Sie eine Antwort aus.*

- immer
- gelegentlich
- ja, aber nur in Ausnahmefällen
- in der Regel nicht

<u>Onkologie – 17:</u>

Bei welchen echokardiographischen Kriterien gehen Sie von einer relevanten Kardiotoxizität aus?

*Bitte wählen eine Antwort aus.*

- LV-EF Abfall > 40%
- LV-EF Abfall > 30%
- LV-EF Abfall > 20%
- LV-EF Abfall > 10%
- jeder LV-EF Abfall stellt eine Kardiotoxizität dar

<u>Onkologie – 18:</u>

Bestimmen Sie NT-ProBNP oder Troponin und wenn ja, bei wie viel Prozent der behandelten Patienten vor einer Chemotherapie haben Sie selbst NT-ProBNP oder Troponin bestimmt?

*Bitte wählen Sie die zutreffende(n) Antwort(en) aus.*

*Bitte geben Sie ggf. den Anteil an behandelten Patienten (in Prozent) an, bei welchem Sie selbst vor einer Chemotherapie NT-ProBNP oder Troponin bestimmt haben.*

- nein, es wird kein NT-ProBNP oder Troponin bestimmt
- ja, es wird NT-ProBNP bestimmt [offene Eingabe]
- ja, es wird Troponin bestimmt [offene Eingabe]

<u>Onkologie – 19:</u>

Was sind die Gründe, warum diese Werte nicht bestimmt werden?

*Bitte wählen Sie die zutreffende(n) Antwort(en) aus.*

- zu hohe Kosten
- wenig Expertise bei der Interpretation der Ergebnisse
- das sollte Aufgabe des Kardiologen sein
- sonstiges [offene Eingabe]

Onkologie – 20:

Gehen Sie wieder von einer Patientin aus, die eine Anthrazyklin Chemotherapie bekommt. Eine Herzinsuffizienz ist bereits bekannt. Wie häufig bestimmen Sie diese Herzwerte?

*Bitte wählen Sie die zutreffende(n) Antwort(en) aus.*

- vor Einleitung der Chemotherapie
- nach jedem Zyklus der Therapie
- nach jedem 2. Zyklus der Therapie
- nach Abschluss der Chemotherapie
- 3 Monate nach Abschluss der Therapie
- 6 Monate nach Abschluss der Therapie
- 12 Monate nach Abschluss der Therapie
- 24 Monate nach Abschluss der Therapie
- bei akuten kardialen Beschwerden
- sonstiges [offene Eingabe]

Onkologie – 21:

Kennen und verwenden Sie kardioprotektive Substanzen in der Behandlung onkologischer Patienten?

*Bitte wählen Sie eine Antwort aus.*

- ja, ich kenne und verwende kardioprotektive Substanzen in der Behandlung onkologischer Patienten
- ich kenne kardioprotektive Substanzen in der Behandlung onkologischer Patienten, aber verwende keine kardioprotektiven
- Substanzen in der Behandlung onkologischer Patienten
- nein, ich kenne und verwende keine kardioprotektiven Substanzen in der Behandlung onkologischer Patienten

Onkologie – 22:

Welche kardioprotektiven Substanzen kennen und verwenden Sie bei Ihren Patienten?

*Bitte wählen Sie die zutreffende(n) Antwort(en) aus.*

- ACE-Hemmer (z.B. Enalapril)
- Beta-Blocker (z.B. Carvedilol)

- Aldosteron Antagonisten (z.B. Spironolacton)
- Statine (z.B. Simvastatin)
- Eisenchelatoren (z.B. Dexrazoxane)
- Thromboyztenaggregationshemmer (z.B. Aspirin)
- Antikoagulantien (z.B. Enoxaparin, Rivaroxaban)
- Neprilysin Inhibitoren
- sonstige [offene Eingabe]

<u>Onkologie – 23:</u>

Wer initiiert die kardioprotektive Behandlung in der Regel bei diesen Patienten?

*Bitte wählen Sie eine Antwort aus.*

- der betreuende Kardiologe
- der Hausarzt
- ich selbst
- ich selbst auf Empfehlung des Kardiologen
- sonstige

<u>Onkologie – 24:</u>

Wie zufrieden sind Sie mit der kardiologischen Betreuung Ihrer Patienten?

*Bitte wählen Sie eine Antwort aus.*

*1 – gering*

*10 – sehr zufrieden*

- [Skala von 1 bis 10]

<u>Onkologie – 25:</u>

Was sind Gründe, warum Sie unzufrieden sind?

*Bitte wählen Sie die zutreffende(n) Antwort(en) aus.*

*Überspringen Sie bitte diese Frage, wenn Sie bei der Frage zuvor 9 oder 10 (sehr zufrieden) ausgewählt haben.*

- fehlende engmaschige Betreuung
- zu wenig kardiologische Konsultationen

- wenig Dialog/Rückmeldung bei Hochrisikopatienten
- keine klaren Aussagen/Festlegungen zum kardiotoxischen Risiko
- sonstiges [offene Eingabe]

<u>Onkologie – 26:</u>

Analog zu verschiedenen Boards in einer Klinik, gibt es in Ihrer Klinik ein regelmäßiges kardioonkologisches Board?

Wenn ja, wie häufig findet dieses statt?

*Bitte wählen Sie eine Antwort aus*

- nein
- ja, täglich
- ja, 1 Mal pro Woche
- ja, 2 Mal pro Monat
- ja, monatlich
- ja, 1 Mal pro Quartal
- sonstiges [offene Eingabe]

<u>Onkologie – 27:</u>

Halten Sie kardioonkologische Boards für sinnvoll?

*Bitte wählen Sie eine Antwort aus.*

- ja, sehr sinnvoll für jeden Patienten
- ja, aber nur für Hochrisikopatienten
- nein, die therapeutische Konsequenz wäre zu gering

<u>Onkologie – 28:</u>

Wie kann die Betreuung kardioonkologischer Patienten aus Ihrer Sicht verbessert werden?

*Bitte wählen Sie die zutreffende(n) Antwort(en) aus.*

- Simplifizierung der Leitlinien
- digitale Tools zur Risikostratifizierung
- Tools zur besseren Bestimmung der Kardiotoxizität
- engere Kommunikation mit behandelnden Kardiologen

- regelmäßige interdisziplinäre Fortbildungen
- Kontaktmöglichkeit zu kardioonkologischem Expertennetzwerk bei schwierigen Fragestellungen
- Tools zur Überwachung des kardialen Status
- sonstiges [offene Eingabe]

***Innere Medizin***

Innere Medizin – 1:

Wo sind Sie tätig?

*Bitte wählen Sie eine Antwort aus.*

- Universitätsklinik
- Universitätsklinik mit dezidierter kardioonkologischer Abteilung
- Akademisches Lehrkrankenhaus
- Maximalversorger
- Grund- oder Regelversorger

Innere Medizin – 2:

Wie viele Patienten mit Chemotherapie werden Ihnen zur Diagnostik/kardiologischen Mitbetreuung durchschnittlich

*monatlich vorgestellt?*

*Bitte geben Sie einen Zahlenwert an.*

- [offene Eingabe]

Innere Medizin – 3:

Wie viel Prozent dieser Patienten entfallen auf die unten aufgeführten Tumorentitäten?

*Bitte geben Sie schätzungsweise einen Wert für den Anteil der genannten Erkrankungen an der Gesamtzahl Ihrer Patienten mit Krebs an.*

- Brustkrebs [Prozentwert zwischen 0 bis 100]
- Prostatakrebs [Prozentwert zwischen 0 bis 100]
- Darmkrebs [Prozentwert zwischen 0 bis 100]
- Lungenkrebs [Prozentwert zwischen 0 bis 100]
- Harnblasenkrebs [Prozentwert zwischen 0 bis 100]

- Nierenkrebs [Prozentwert zwischen 0 bis 100]
- Krebs von Mundhöhle und Rachen [Prozentwert zwischen 0 bis 100]
- Leukämien [Prozentwert zwischen 0 bis 100]
- Non-Hodgkin-Lymphome [Prozentwert zwischen 0 bis 100]
- sonstige Krebserkrankungen [Prozentwert zwischen 0 bis 100]

Innere Medizin – 4:

Wie viel Prozent sind ambulante Patienten?

*Bitte geben Sie schätzungsweise einen Wert an.*

- Anteil ambulanter Patienten [Prozentwert zwischen 0 bis 100]

Innere Medizin – 5:

In welchen Schritten der Behandlung von Patienten mit potentiell kardiotoxischer Chemotherapie sind Sie involviert?

*Bitte wählen Sie die zutreffende(n) Antwort(en) aus.*

- kardiotoxische Risikostratifizierung der Chemotherapie
- kardiologische Diagnostik vor der Chemotherapie
- kardiologische Diagnostik während der Chemotherapie
- kardiologische Diagnostik nach der Chemotherapie
- Initiierung der Therapie bei eingetretener Kardiotoxizität
- Anpassung der Therapie bei eingetretener Kardiotoxizität
- Tumorboards

Innere Medizin – 6:

Führen Sie eine Stratifizierung des potentiell kardiotoxischen Risikos der Chemotherapie durch?

*Bitte wählen Sie eine Antwort aus.*

- ja, allein
- ja, aber in Zusammenarbeit mit einem interdisziplinären Team
- nein
- nein, das ist generell die Aufgabe des betreuenden fachspezifischen Onkologen/Hämatologen

Innere Medizin – 7:

Hat die von Ihnen durchgeführte Risikoerhebung des kardiotoxischen Risikos einen Einfluss auf die Auswahl der Chemotherapie der Patienten?

*Bitte wählen Sie eine Antwort aus.*

- ja, ich gebe allein dezidierte Empfehlungen zur Anpassung der Therapie
- ja, ggf. konsultiere ich den Onkologen/Gynäkologen und das kardiotoxische Risiko wird für jeden Patienten gemeinsam evaluiert
- nein, es erfolgt keinerlei Kommunikation

Innere Medizin – 8:

Welche Parameter fließen in die Risikobewertung des kardiotoxischen Risikos mit ein?

*Bitte wählen Sie die zutreffende(n) Antwort(en) aus.*

- kardiale Vorerkrankungen (z.B. Herzinsuffizienz, KHK)
- Alter (>60 Jahre)
- Geschlecht
- Diabetes mellitus
- Niereninsuffizienz
- Übergewicht
- hohe Dosis der Anthrazyklin Chemotherapie
- zusätzliche Radiotherapie, wobei das Herz im Bestrahlungsfeld ist
- HER2 Inhibitoren, Proteasom Inhibitoren, VEGF Therapie
- erhöhte kardiale Biomarker
- linksventrikuläre Pumpfunktion
- sonstige [offene Eingabe]

Innere Medizin – 9:

Welche Untersuchungen führen Sie standardmäßig bei diesen Patienten durch?

*Bitte wählen Sie die zutreffende(n) Antwort(en) aus.*

- Anamnese
- EKG

- Echokardiographie
- Ergometrie
- Labor TNT
- Labor BNP
- Laborparameter wie z.B. Blutglukose, GFR, Lipidprofil
- sonstiges [offene Eingabe]

Innere Medizin – 10:

Gehen Sie von einer Patientin aus, die eine Anthrazyklin Chemotherapie bekommt. Eine Herzinsuffizienz ist bereits bekannt.

Wie oft wird Ihnen diese Patientin vorgestellt?

*Bitte wählen Sie die zutreffende(n) Antwort(en) aus.*

- vor Einleitung der Chemotherapie
- nach jedem Zyklus der Therapie
- nach jedem 2. Zyklus der Therapie
- nach Abschluss der Chemotherapie
- 3 Monate nach Abschluss der Therapie
- 6 Monate nach Abschluss der Therapie
- 12 Monate nach Abschluss der Therapie
- 24 Monate nach Abschluss der Therapie
- bei akuten kardialen Beschwerden
- sonstiges [offene Eingabe]

Innere Medizin – 11:

Werden Sie bei Patienten mit vermuteter chemotherapiebedingter Kardiotoxizität in die *weitere Abwägung der Fortführung/Anpassung der Chemotherapie mit einbezogen?*

*Bitte wählen Sie eine Antwort aus.*

- immer
- gelegentlich
- ja, aber nur in Ausnahmefällen

- nein

Innere Medizin – 12:

Bei welchen echokardiographischen Kriterien gehen Sie von einer relevanten Kardiotoxizität aus und kommunizieren das?

*Bitte wählen Sie eine Antwort aus.*

- LV-EF Abfall > 40% zu einem Wert unterhalb der Grenzwerte
- LV-EF Abfall > 30% zu einem Wert unterhalb der Grenzwerte
- LV-EF Abfall > 20% zu einem Wert unterhalb der Grenzwerte
- LV-EF Abfall > 10% zu einem Wert unterhalb der Grenzwerte
- jeder LV-EF Abfall stellt eine Kardiotoxizität dar

Innere Medizin – 13:

Nutzen Sie neben der LV-EF Bestimmung regelhaft echokardiographische Strainuntersuchungen zur kardialen Verlaufskontrolle nach Chemotherapie?

*Bitte wählen Sie eine Antwort aus.*

- ja
- nein

Innere Medizin – 14:

Was sind Gründe, warum keine Strainuntersuchungen stattfinden?

*Bitte wählen Sie die zutreffende(n) Antwort(en) aus.*

- fehlende technische Ausrüstung
- zu zeitaufwendig
- Schallbedingungen oft nicht ausreichend
- therapeutische Konsequenz unklar
- fehlende Expertise
- fehlendes Personal
- sonstiges [offene Eingabe]

Innere Medizin – 15:

Bestimmen Sie NT-ProBNP oder Troponin und wenn ja, bei wie viel Prozent der behandelten Patienten vor einer Chemotherapie haben Sie selbst NT-ProBNP oder Troponin bestimmt?

*Bitte wählen Sie die zutreffende(n) Antwort(en) aus.*

*Bitte geben Sie ggf. den Anteil an behandelten Patienten (in Prozent) an, bei welchem Sie selbst vor einer Chemotherapie NT-ProBNP oder Troponin bestimmt haben.*

- nein, es wird kein NT-ProBNP oder Troponin bestimmt
- ja, es wird NT-ProBNP bestimmt [offene Eingabe]
- ja, es wird Troponin bestimmt [offene Eingabe]

Innere Medizin – 16:

Gehen Sie wieder von einer Patientin aus, die eine Anthrazyklin Chemotherapie bekommt. Eine Herzinsuffizienz ist bereits bekannt. Wie häufig bestimmen Sie diese Herzwerte?

*Bitte wählen Sie die zutreffende(n) Antwort(en) aus.*

- vor Einleitung der Chemotherapie
- nach jedem Zyklus der der Therapie
- nach jedem 2. Zyklus der Therapie
- nach Abschluss der Chemotherapie
- 3 Monate nach Abschluss der Therapie
- 6 Monate nach Abschluss der Therapie
- 12 Monate nach Abschluss der Therapie
- bei akuten kardialen Beschwerden
- bei: [offene Eingabe]
- nein, es werden keine Werte bestimmt

Innere Medizin – 17:

Was sind die Gründe, warum diese Werte nicht bestimmt werden?

*Bitte wählen Sie die zutreffende(n) Antwort(en) aus.*

- Das sollte Aufgabe des Onkologen sein.
- zu hohe Kosten
- Die therapeutische Konsequenz ist unklar.
- wenig Expertise bei der Interpretation der Ergebnisse
- sonstiges [offene Eingabe]

Innere Medizin – 18:

Kennen und verwenden Sie kardioprotektive Substanzen in der Behandlung onkologischer Patienten?

*Bitte wählen Sie eine Antwort aus.*

- ja, ich kenne und verwende kardioprotektive Substanzen in der Behandlung onkologischer Patienten
- ich kenne kardioprotektive Substanzen in der Behandlung onkologischer Patienten, aber verwende keine kardioprotektiven
- Substanzen in der Behandlung onkologischer Patienten
- nein, ich kenne und verwende keine kardioprotektiven Substanzen in der Behandlung onkologischer Patienten

Innere Medizin – 19:

Welche Substanzen kennen und verwenden Sie bei Ihren Patienten?

*Bitte wählen Sie die zutreffende(n) Antwort(en) aus.*

- ACE-Hemmer (z.B. Enalapril)
- Beta-Blocker (z.B. Carvedilol)
- ARNI (z.B. Entresto)
- Aldosteron Antagonisten (z.B. Spironolacton)
- Statine (z.B. Atorvastatin)
- Eisenchelatoren (z.B. Dexrazoxan)
- Thrombozytenaggregationshemmer (z.B. Aspirin)
- Antikoagulantien (z.B. Enoxaparin, Rivaroxaban)
- Neprilysin Inhibitoren
- sonstige [offene Eingabe]

Innere Medizin – 20:

Wer initiiert die kardioprotektive Behandlung in der Regel bei diesen Patienten?

*Bitte wählen Sie eine Antwort aus.*

- der betreuende Onkologe
- der ambulante Kardiologe
- der Hausarzt

- ich selbst
- ich formuliere lediglich eine Empfehlung
- sonstige [offene Eingabe]

Innere Medizin – 21:

Gibt es zwischen Ihnen und den betreuenden Onkologen einen engen Dialog über die Ergebnisse der kardialen Diagnostik und die Implikationen auf die daraus resultierende Therapie (sowohl kardiologisch und onkologisch)?

*Bitte wählen Sie eine Antwort aus.*

- ja, es besteht immer ein aktiver Dialog ggf. auch telefonisch, welcher auch Einfluss auf die Therapie hat
- ja, es besteht ein aktiver Dialog ggf. auch telefonisch bei bestimmen Risikopatienten, welcher auch Einfluss auf die Therapie hat
- nein, ich gebe dezidierte therapeutische Ratschläge im Befundbrief
- nein, alle wesentlichen Informationen sollten die behandelnden Onkologen/Gynäkologen aus der durchgeführten Diagnostik
- selbst interpretieren
- sonstiges [offene Eingabe]

Innere Medizin – 22:

Ist aus Ihrer Sicht eine spezialisierte kardioonkologische Betreuung von Patienten mit Chemotherapie sinnvoll?

*Bitte wählen Sie eine Antwort aus.*

*Bitte geben Sie ggf. eine Begründung an.*

- ja [offene Eingabe]
- nein [offene Eingabe]

Innere Medizin – 23:

Analog zu verschiedenen Boards in einer Klinik, gibt es in Ihrer Klinik ein regelmäßiges kardioonkologisches Board?

Wenn ja wie häufig findet dieses statt?

*Bitte wählen Sie Antwort aus.*

*Bitte geben Sie ggf. die Häufigkeit an.*

- ja [offene Eingabe]

- nein

Innere Medizin – 24:

Halten Sie kardioonkologische Boards für sinnvoll?

*Bitte wählen Sie ein Antwort aus.*

- ja, sehr sinnvoll für jeden Patienten
- ja, aber nur sinnvoll für Hochrisikopatienten
- nein, die therapeutische Konsequenz wäre zu gering

Innere Medizin – 25:

Wie schätzen Sie die interdisziplinäre Betreuung kardioonkologischer Patienten bei Ihnen in der Klinik ein?

*Bitte geben Sie an wie viel Prozent bis zur optimalen Betreuung fehlen.*

- Prozent bis zur optimalen Betreuung [Prozentwert zwischen 0 bis 100]

Innere Medizin – 26:

Wie kann die Betreuung kardioonkologischer Patienten aus Ihrer Sicht verbessert werden?

*Bitte wählen Sie die zutreffende(n) Antwort(enI aus.*

- Simplifizierung der Leitlinien
- digitale Tools zur Risikostratifizierung
- Tools zur besseren Bestimmung der Kardiotoxizität
- engere Kommunikation mit behandelnden Onkologen
- regelmäßige interdisziplinäre Fortbildungen
- Kontaktmöglichkeit zu kardioonkologischem Expertennetzwerk bei schwierigen Fragestellungen
- Tools zur Überwachung des kardialen Status
- sonstiges

## Survey questionnaire – English translation

*English translation of the questionnaire reproduced above, provided for reference. The survey was administered in German; specialty-specific skip logic was applied so that each respondent completed only the section corresponding to their department (Gynaecology, Internal Medicine, Cardiology or Oncology).*

### Inpatient cardio-oncology questionnaire

In what type of department do you work?

*Please select one answer.*

- Gynaecology
- Internal medicine
- Cardiology
- Oncology

***Gynaecology***

<u>Gynaecology – 1:</u>

Where do you work?

*Please select one answer.*

- University hospital
- University hospital with a dedicated cardio-oncology unit
- academic teaching hospital
- maximum-care hospital
- primary or standard-care hospital

<u>Gynaecology – 2:</u>

Approximately how many patients with cancer do you treat with cardiotoxic chemotherapy per month?

*Please enter a numeric value.*

- [free-text entry]

<u>Gynaecology – 3:</u>

What percentage of these patients falls into the tumour entities listed below?

*Please provide an approximate value for the proportion of the listed diseases among the total number of your cancer patients.*

- Breast cancer [percentage between 0 and 100]
- Uterine (corpus) cancer [percentage between 0 and 100]
- Cancer of the ovaries and adnexa [percentage between 0 and 100]
- other cancers [percentage between 0 and 100]

Gynaecology – 4:

What percentage are outpatients?

*Please provide an approximate value.*

- proportion of outpatients [percentage between 0 and 100]

Gynaecology – 5:

What proportion of your cancer patients receiving cardiotoxic chemotherapy (anthracyclines, HER2 receptor antagonists, etc.) are under cardiological co-management?

*Please enter a percentage.*

- [free-text entry]

Gynaecology – 6:

What are possible reasons why not all patients receive cardiological co-management?

*Please select the applicable answer(s).*

- no indication for cardiological co-management
- lack of cardiological capacity
- lack of therapeutic consequences
- Dialogue with the cardiologists is insufficient to derive therapeutic consequences from it.
- other [free-text entry]

Gynaecology – 7:

How is the cardiological co-management of patients provided?

*Please provide an approximate ratio.*

*The midpoint of the scale corresponds to a ratio of 50% outpatient and 50% inpatient co-management.*

- ratio of outpatient to inpatient co-management [percentage between 0 and 100]

Gynaecology – 8:

At which steps in the treatment of patients receiving potentially cardiotoxic chemotherapy do you involve cardiologists?

*Please select the applicable answer(s).*

- cardiotoxic-risk stratification of chemotherapy
- cardiological diagnostics before chemotherapy
- cardiological diagnostics during chemotherapy
- cardiological diagnostics after chemotherapy
- adjustment of therapy when cardiotoxicity has occurred
- other [free-text entry]

<u>Gynaecology – 9:</u>

Do you perform cardiotoxic-risk stratification before starting chemotherapy?

*Please select one answer.*

- yes
- no

<u>Gynaecology – 10:</u>

Which parameters do you include in the assessment of cardiotoxic risk?

*Please select the applicable answer(s).*

- pre-existing cardiac disease (e.g. heart failure, coronary artery disease)
- age (>60 years)
- sex
- diabetes mellitus
- renal insufficiency
- obesity
- high-dose anthracycline chemotherapy
- additional radiotherapy with the heart in the radiation field
- HER2 inhibitors, proteasome inhibitors, VEGF therapy
- elevated cardiac biomarkers
- left ventricular function

- other [free-text entry]

Gynaecology – 11:

If a cardiotoxic-risk assessment is performed, does it influence the choice of chemotherapy?

*Please select one answer*.

- no
- yes, I adjust the chemotherapy on my own
- yes, if needed I consult a cardiologist and the cardiotoxic risk is jointly evaluated for each patient and the therapy is discussed

Gynaecology – 12:

What are the reasons why you do not perform risk stratification?

[multiple selection possible]

- This is the task of the treating cardiologist.
- There is a lack of time to perform this assessment.
- There is a lack of the necessary instruments to perform this assessment.
- Interpretation of the results is too complex.
- The assessment is too costly.
- other [free-text entry]

Gynaecology – 13:

Which examinations are performed by the cardiologist at your request?

*Please select the applicable answer(s).*

- history
- ECG
- echocardiography
- ergometry
- laboratory: troponin T (TnT)
- laboratory: BNP
- laboratory parameters such as blood glucose, GFR, lipid profile
- other [free-text entry]

Gynaecology – 14:

Consider a patient receiving anthracycline chemotherapy who already has known heart failure. How often is this patient referred for cardiological consultation?

*Please select the applicable answer(s).*

- before initiation of chemotherapy
- after every cycle of therapy
- after every second cycle of therapy
- after completion of chemotherapy
- 3 months after completion of therapy
- 6 months after completion of therapy
- 12 months after completion of therapy
- 24 months after completion of therapy
- in the event of acute cardiac symptoms
- other [free-text entry]

Gynaecology – 15:

Is there close dialogue between you and the treating cardiologists regarding the results of cardiac diagnostics and their implications for the resulting therapy (both cardiological and oncological)?

*Please select one answer.*

- yes, there is always an active dialogue, if needed also by telephone, which also influences therapy
- yes, there is an active dialogue, if needed also by telephone, for certain high-risk patients, which also influences therapy
- no, I receive specific therapeutic advice only in the written report
- no, I have to interpret all essential information from the diagnostics myself
- other [free-text entry]

Gynaecology – 16:

For patients with suspected chemotherapy-related cardiotoxicity, do you involve cardiologists in the further decision on continuing/adjusting chemotherapy?

*Please select one answer.*

- always

- occasionally
- yes, but only in exceptional cases
- usually not

Gynaecology – 17:

At which echocardiographic criteria do you assume relevant cardiotoxicity?

*Please select one answer.*

- LVEF decline > 40%
- LVEF decline > 30%
- LVEF decline > 20%
- LVEF decline > 10%
- any LVEF decline represents cardiotoxicity

Gynaecology – 18:

Do you measure NT-proBNP or troponin, and if so, in what percentage of treated patients did you yourself measure NT-proBNP or troponin before chemotherapy?

*Please select the applicable answer(s).*

*Please indicate, if applicable, the proportion of treated patients (in percent) in whom you yourself measured NT-proBNP or troponin before chemotherapy.*

- no, neither NT-proBNP nor troponin is measured
- yes, NT-proBNP is measured [free-text entry]
- yes, troponin is measured [free-text entry]

Gynaecology – 19:

What are the reasons why these values are not measured?

*Please select the applicable answer(s).*

- costs too high
- little expertise in interpreting the results
- this should be the task of the cardiologist
- other [free-text entry]

Gynaecology – 20:

Again consider a patient receiving anthracycline chemotherapy who already has known heart failure. How often do you measure these cardiac values?

*Please select the applicable answer(s).*

- before initiation of chemotherapy
- after every cycle of therapy
- after every second cycle of therapy
- after completion of chemotherapy
- 3 months after completion of therapy
- 6 months after completion of therapy
- 12 months after completion of therapy
- 24 months after completion of therapy
- in the event of acute cardiac symptoms
- other [free-text entry]

<u>Gynaecology – 21:</u>

Do you know and use cardioprotective agents in the treatment of oncology patients?

*Please select one answer.*

- yes, I know and use cardioprotective agents in the treatment of oncology patients
- I know cardioprotective agents in the treatment of oncology patients but do not use any cardioprotective
- agents in the treatment of oncology patients
- no, I do not know or use any cardioprotective agents in the treatment of oncology patients

<u>Gynaecology – 22:</u>

Which cardioprotective agents do you know and use in your patients?

*Please select the applicable answer(s).*

- ACE inhibitors (e.g. enalapril)
- beta-blockers (e.g. carvedilol)
- aldosterone antagonists (e.g. spironolactone)
- statins (e.g. simvastatin)

- iron chelators (e.g. dexrazoxane)
- antiplatelet agents (e.g. aspirin)
- anticoagulants (e.g. enoxaparin, rivaroxaban)
- neprilysin inhibitors
- other [free-text entry]

Gynaecology – 23:

Who usually initiates cardioprotective treatment in these patients?

- *Please select one answer.*
- the treating cardiologist
- the general practitioner
- myself
- myself, on the cardiologist's recommendation
- other [free-text entry]

Gynaecology – 24:

How satisfied are you with the cardiological care of your patients?

*Please select one answer.*

*1 – low*

*10 – very satisfied*

- [scale from 1 to 10]

Gynaecology – 25:

What are the reasons for your dissatisfaction?

*Please select the applicable answer(s).*

*Please skip this question if you selected 9 or 10 (very satisfied) in the previous question.*

- lack of close monitoring
- too few cardiological consultations
- little dialogue/feedback for high-risk patients
- no clear statements/determinations on cardiotoxic risk
- other [free-text entry]

Gynaecology – 26:

Analogous to the various boards in a hospital, is there a regular cardio-oncology board at your institution?

If yes, how often does it take place?

*Please select one answer*

- no
- yes, daily
- yes, once a week
- yes, twice a month
- yes, monthly
- yes, once a quarter
- other [free-text entry]

Gynaecology – 27:

Do you consider cardio-oncology boards useful?

*Please select one answer*.

- yes, very useful for every patient
- yes, but only for high-risk patients
- no, the therapeutic consequence would be too small

Gynaecology – 28:

In your view, how can the care of cardio-oncology patients be improved?

*Please select the applicable answer(s).*

- simplification of the guidelines
- digital tools for risk stratification
- tools for better determination of cardiotoxicity
- closer communication with treating cardiologists
- regular interdisciplinary continuing education
- option to contact a cardio-oncology expert network for difficult questions
- tools for monitoring cardiac status

- other

***Cardiology***

Cardiology – 1:

Where do you work?

*Please select one answer.*

- University hospital
- University hospital with a dedicated cardio-oncology unit
- academic teaching hospital
- maximum-care hospital
- primary or standard-care hospital

Cardiology – 2:

On average, how many chemotherapy patients are referred to you per month for diagnostics/cardiological co-management?

*Please enter a numeric value.*

- [free-text entry]

Cardiology – 3:

What percentage of these patients falls into the tumour entities listed below?

*Please provide an approximate value for the proportion of the listed diseases among the total number of your cancer patients.*

- Breast cancer [percentage between 0 and 100]
- Prostate cancer [percentage between 0 and 100]
- Colorectal cancer [percentage between 0 and 100]
- Lung cancer [percentage between 0 and 100]
- Bladder cancer [percentage between 0 and 100]
- Kidney cancer [percentage between 0 and 100]
- Cancer of the oral cavity and pharynx [percentage between 0 and 100]
- Leukaemias [percentage between 0 and 100]
- Non-Hodgkin lymphomas [percentage between 0 and 100]

- other cancers [percentage between 0 and 100]

Cardiology – 4:

What percentage are outpatients?

*Please provide an approximate value.*

- proportion of outpatients [percentage between 0 and 100]

Cardiology – 5:

At which steps in the treatment of patients receiving potentially cardiotoxic chemotherapy are you involved?

*Please select the applicable answer(s).*

- cardiotoxic-risk stratification of chemotherapy
- cardiological diagnostics before chemotherapy
- cardiological diagnostics during chemotherapy
- cardiological diagnostics after chemotherapy
- initiation of therapy when cardiotoxicity has occurred
- adjustment of therapy when cardiotoxicity has occurred
- tumour boards

Cardiology – 6:

Do you perform stratification of the potential cardiotoxic risk of chemotherapy?

*Please select one answer.*

- yes, on my own
- yes, but in collaboration with an interdisciplinary team
- no
- no, this is generally the task of the treating specialist oncologist/haematologist

Cardiology – 7:

Does the cardiotoxic-risk assessment you perform influence the choice of the patients' chemotherapy?

*Please select one answer.*

- yes, I give specific recommendations for adjusting therapy on my own

- yes, if needed I consult the oncologist/gynaecologist and the cardiotoxic risk is jointly evaluated for each patient
- no, there is no communication at all

<u>Cardiology – 8:</u>

Which parameters do you include in the assessment of cardiotoxic risk?

*Please select the applicable answer(s).*

- pre-existing cardiac disease (e.g. heart failure, coronary artery disease)
- age (>60 years)
- sex
- diabetes mellitus
- renal insufficiency
- obesity
- high-dose anthracycline chemotherapy
- additional radiotherapy with the heart in the radiation field
- HER2 inhibitors, proteasome inhibitors, VEGF therapy
- elevated cardiac biomarkers
- left ventricular function
- other [free-text entry]

<u>Cardiology – 9:</u>

Which examinations do you routinely perform in these patients?

*Please select the applicable answer(s).*

- history
- ECG
- echocardiography
- ergometry
- laboratory: troponin T (TnT)
- laboratory: BNP
- laboratory parameters such as blood glucose, GFR, lipid profile

- other [free-text entry]

Cardiology – 10:

Consider a patient receiving anthracycline chemotherapy who already has known heart failure.

How often is this patient referred to you?

*Please select the applicable answer(s).*

- before initiation of chemotherapy
- after every cycle of therapy
- after every second cycle of therapy
- after completion of chemotherapy
- 3 months after completion of therapy
- 6 months after completion of therapy
- 12 months after completion of therapy
- 24 months after completion of therapy
- in the event of acute cardiac symptoms
- other [free-text entry]

Cardiology – 11:

For patients with suspected chemotherapy-related cardiotoxicity, are you involved in the further decision on continuing/adjusting chemotherapy?

*Please select one answer.*

- always
- occasionally
- yes, but only in exceptional cases
- no

Cardiology – 12:

At which echocardiographic criteria do you assume relevant cardiotoxicity and communicate this?

*Please select one answer.*

- LVEF decline > 40% to a value below the reference limits

- LVEF decline > 30% to a value below the reference limits
- LVEF decline > 20% to a value below the reference limits
- LVEF decline > 10% to a value below the reference limits
- any LVEF decline represents cardiotoxicity

Cardiology – 13:

In addition to LVEF measurement, do you routinely use echocardiographic strain analysis for cardiac follow-up after chemotherapy?

*Please select one answer*.

- yes
- no

Cardiology – 14:

What are the reasons why strain analyses are not performed?

*Please select the applicable answer(s).*

- lack of technical equipment
- too time-consuming
- acoustic windows often insufficient
- therapeutic consequence unclear
- lack of expertise
- lack of personnel
- other [free-text entry]

Cardiology – 15:

Do you measure NT-proBNP or troponin, and if so, in what percentage of treated patients did you yourself measure NT-proBNP or troponin before chemotherapy?

*Please select the applicable answer(s).*

*Please indicate, if applicable, the proportion of treated patients (in percent) in whom you yourself measured NT-proBNP or troponin before chemotherapy.*

- no, neither NT-proBNP nor troponin is measured
- yes, NT-proBNP is measured [free-text entry]
- yes, troponin is measured [free-text entry]

Cardiology – 16:

Again consider a patient receiving anthracycline chemotherapy who already has known heart failure. How often do you measure these cardiac values?

*Please select the applicable answer(s).*

- before initiation of chemotherapy
- after every cycle of therapy
- after every second cycle of therapy
- after completion of chemotherapy
- 3 months after completion of therapy
- 6 months after completion of therapy
- 12 months after completion of therapy
- in the event of acute cardiac symptoms
- for: [free-text entry]
- no, no values are measured

Cardiology – 17:

What are the reasons why these values are not measured?

*Please select the applicable answer(s).*

- This should be the task of the oncologist.
- costs too high
- The therapeutic consequence is unclear.
- little expertise in interpreting the results
- other [free-text entry]

Cardiology – 18:

Do you know and use cardioprotective agents in the treatment of oncology patients?

*Please select one answer.*

- yes, I know and use cardioprotective agents in the treatment of oncology patients
- I know cardioprotective agents in the treatment of oncology patients but do not use any cardioprotective

- agents in the treatment of oncology patients
- no, I do not know or use any cardioprotective agents in the treatment of oncology patients

Cardiology – 19:

Which agents do you know and use in your patients?

*Please select the applicable answer(s).*

- ACE inhibitors (e.g. enalapril)
- beta-blockers (e.g. carvedilol)
- ARNI (e.g. Entresto)
- aldosterone antagonists (e.g. spironolactone)
- statins (e.g. atorvastatin)
- iron chelators (e.g. dexrazoxane)
- antiplatelet agents (e.g. aspirin)
- anticoagulants (e.g. enoxaparin, rivaroxaban)
- neprilysin inhibitors
- other [free-text entry]

Cardiology – 20:

Who usually initiates cardioprotective treatment in these patients?

*Please select one answer.*

- the treating oncologist
- the outpatient cardiologist
- the general practitioner
- myself
- I only formulate a recommendation
- other [free-text entry]

Cardiology – 21:

Is there close dialogue between you and the treating oncologists regarding the results of cardiac diagnostics and their implications for the resulting therapy (both cardiological and oncological)?

*Please select one answer.*

- yes, there is always an active dialogue, if needed also by telephone, which also influences therapy
- yes, there is an active dialogue, if needed also by telephone, for certain high-risk patients, which also influences therapy
- no, I give specific therapeutic advice in the written report
- no, the treating oncologists/gynaecologists should interpret all essential information from the diagnostics
- themselves
- other [free-text entry]

<u>Cardiology – 22:</u>

In your view, is specialised cardio-oncology care useful for chemotherapy patients?

*Please select one answer.*

*Please provide a reason, if applicable.*

- yes [free-text entry]
- no [free-text entry]

<u>Cardiology – 23:</u>

Analogous to the various boards in a hospital, is there a regular cardio-oncology board at your institution?

If yes, how often does it take place?

*Please select one answer.*

*Please indicate the frequency, if applicable.*

- yes [free-text entry]
- no

<u>Cardiology – 24:</u>

Do you consider cardio-oncology boards useful?

*Please select one answer.*

- yes, very useful for every patient
- yes, but only useful for high-risk patients
- no, the therapeutic consequence would be too small

Cardiology – 25:

How do you rate the interdisciplinary care of cardio-oncology patients at your institution?

*Please indicate what percentage is missing to reach optimal care.*

- percentage to optimal care [percentage between 0 and 100]

Cardiology – 26:

In your view, how can the care of cardio-oncology patients be improved?

*Please select the applicable answer(s).*

- simplification of the guidelines
- digital tools for risk stratification
- tools for better determination of cardiotoxicity
- closer communication with treating oncologists
- regular interdisciplinary continuing education
- option to contact a cardio-oncology expert network for difficult questions
- tools for monitoring cardiac status
- other [free-text entry]

***Oncology***

Oncology – 1:

Where do you work?

*Please select one answer.*

- University hospital
- University hospital with a dedicated cardio-oncology unit
- academic teaching hospital
- maximum-care hospital
- primary or standard-care hospital

Oncology – 2:

Approximately how many patients with cancer do you treat with cardiotoxic chemotherapy per month?

*Please enter a numeric value.*

- [free-text entry]

<u>Oncology – 3:</u>

What percentage of these patients falls into the tumour entities listed below?

*Please provide an approximate value for the proportion of the listed diseases among the total number of your cancer patients.*

- Prostate cancer [percentage between 0 and 100]
- Colorectal cancer [percentage between 0 and 100]
- Lung cancer [percentage between 0 and 100]
- Bladder cancer [percentage between 0 and 100]
- Kidney cancer [percentage between 0 and 100]
- Cancer of the oral cavity and pharynx [percentage between 0 and 100]
- Leukaemias [percentage between 0 and 100]
- Non-Hodgkin lymphomas [percentage between 0 and 100]
- other cancers [percentage between 0 and 100]

<u>Oncology – 4:</u>

What percentage are outpatients?

*Please provide an approximate value.*

- proportion of outpatients [percentage between 0 and 100]

<u>Oncology – 5:</u>

What proportion of your cancer patients receiving cardiotoxic chemotherapy (anthracyclines, HER2 receptor antagonists, etc.) are under cardiological co-management?

*Please enter a percentage.*

- [free-text entry]

<u>Oncology – 6:</u>

What are possible reasons why not all patients receive cardiological co-management?

*Please select the applicable answer(s).*

- no indication for cardiological co-management
- lack of cardiological capacity
- lack of therapeutic consequences

- Dialogue with the cardiologists is insufficient to derive therapeutic consequences from it.
- other [free-text entry]

Oncology – 7:

How is the cardiological co-management of patients provided?

*Please provide an approximate ratio.*

*The midpoint of the scale corresponds to a ratio of 50% outpatient and 50% inpatient co-management.*

- ratio of outpatient to inpatient co-management [percentage between 0 and 100]

Oncology – 8:

At which steps in the treatment of patients receiving potentially cardiotoxic chemotherapy do you involve cardiologists?

*Please select the applicable answer(s).*

- cardiotoxic-risk stratification of chemotherapy
- cardiological diagnostics before chemotherapy
- cardiological diagnostics during chemotherapy
- cardiological diagnostics after chemotherapy
- adjustment of therapy when cardiotoxicity has occurred
- other [free-text entry]

Oncology – 9:

Do you perform cardiotoxic-risk stratification before starting chemotherapy?

*Please select one answer.*

- yes
- no

Oncology – 10:

Which parameters do you include in the assessment of cardiotoxic risk?

*Please select the applicable answer(s).*

- pre-existing cardiac disease (e.g. heart failure, coronary artery disease)
- age (>60 years)
- sex

- diabetes mellitus
- renal insufficiency
- obesity
- high-dose anthracycline chemotherapy
- additional radiotherapy with the heart in the radiation field
- HER2 inhibitors, proteasome inhibitors, VEGF therapy
- elevated cardiac biomarkers
- left ventricular function
- other [free-text entry]

Oncology – 11:

If a cardiotoxic-risk assessment is performed, does it influence the choice of chemotherapy?

*Please select one answer.*

- no
- yes, I adjust the chemotherapy on my own
- yes, if needed I consult a cardiologist and the cardiotoxic risk is jointly evaluated for each patient and the
- therapy is discussed

Oncology – 12:

What are the reasons why you do not perform risk stratification?

[multiple selection possible]

- This is the task of the treating cardiologist.
- There is a lack of time to perform this assessment.
- There is a lack of the necessary instruments to perform this assessment.
- Interpretation of the results is too complex.
- The assessment is too costly.
- other [free-text entry]

Oncology – 13:

*Which examinations are performed by the cardiologist at your request?*

*Please select the applicable answer(s).*

- history
- ECG
- echocardiography
- ergometry
- laboratory: troponin T (TnT)
- laboratory: BNP
- laboratory parameters such as blood glucose, GFR, lipid profile
- other [free-text entry]

<u>Oncology – 14:</u>

Consider a patient receiving anthracycline chemotherapy who already has known heart failure. How often is this patient referred for cardiological consultation?

*Please select the applicable answer(s).*

- before initiation of chemotherapy
- after every cycle of therapy
- after every second cycle of therapy
- after completion of chemotherapy
- 3 months after completion of therapy
- 6 months after completion of therapy
- 12 months after completion of therapy
- 24 months after completion of therapy
- in the event of acute cardiac symptoms
- other [free-text entry]

<u>Oncology – 15:</u>

Is there close dialogue between you and the treating cardiologists regarding the results of cardiac diagnostics and their implications for the resulting therapy (both cardiological and oncological)?

*Please select one answer.*

- yes, there is always an active dialogue, if needed also by telephone, which also influences therapy

- yes, there is an active dialogue, if needed also by telephone, for certain high-risk patients, which also influences therapy
- no, I receive specific therapeutic advice only in the written report
- no, I have to interpret all essential information from the diagnostics myself
- other [free-text entry]

Oncology – 16:

For patients with suspected chemotherapy-related cardiotoxicity, do you involve cardiologists in the further decision on continuing/adjusting chemotherapy?

*Please select one answer.*

- always
- occasionally
- yes, but only in exceptional cases
- usually not

Oncology – 17:

At which echocardiographic criteria do you assume relevant cardiotoxicity?

*Please select one answer.*

- LVEF decline > 40%
- LVEF decline > 30%
- LVEF decline > 20%
- LVEF decline > 10%
- any LVEF decline represents cardiotoxicity

Oncology – 18:

Do you measure NT-proBNP or troponin, and if so, in what percentage of treated patients did you yourself measure NT-proBNP or troponin before chemotherapy?

*Please select the applicable answer(s).*

*Please indicate, if applicable, the proportion of treated patients (in percent) in whom you yourself measured NT-proBNP or troponin before chemotherapy.*

- no, neither NT-proBNP nor troponin is measured
- yes, NT-proBNP is measured [free-text entry]
- yes, troponin is measured [free-text entry]

Oncology – 19:

What are the reasons why these values are not measured?

*Please select the applicable answer(s).*

- costs too high
- little expertise in interpreting the results
- this should be the task of the cardiologist
- other [free-text entry]

Oncology – 20:

Again consider a patient receiving anthracycline chemotherapy who already has known heart failure. How often do you measure these cardiac values?

*Please select the applicable answer(s).*

- before initiation of chemotherapy
- after every cycle of therapy
- after every second cycle of therapy
- after completion of chemotherapy
- 3 months after completion of therapy
- 6 months after completion of therapy
- 12 months after completion of therapy
- 24 months after completion of therapy
- in the event of acute cardiac symptoms
- other [free-text entry]

Oncology – 21:

Do you know and use cardioprotective agents in the treatment of oncology patients?

*Please select one answer.*

- yes, I know and use cardioprotective agents in the treatment of oncology patients
- I know cardioprotective agents in the treatment of oncology patients but do not use any cardioprotective
- agents in the treatment of oncology patients

- no, I do not know or use any cardioprotective agents in the treatment of oncology patients

<u>Oncology – 22:</u>

Which cardioprotective agents do you know and use in your patients?

*Please select the applicable answer(s).*

- ACE inhibitors (e.g. enalapril)
- beta-blockers (e.g. carvedilol)
- aldosterone antagonists (e.g. spironolactone)
- statins (e.g. simvastatin)
- iron chelators (e.g. dexrazoxane)
- antiplatelet agents (e.g. aspirin)
- anticoagulants (e.g. enoxaparin, rivaroxaban)
- neprilysin inhibitors
- other [free-text entry]

<u>Oncology – 23:</u>

Who usually initiates cardioprotective treatment in these patients?

*Please select one answer.*

- the treating cardiologist
- the general practitioner
- myself
- myself, on the cardiologist’s recommendation
- other

<u>Oncology – 24:</u>

How satisfied are you with the cardiological care of your patients?

*Please select one answer.*

*1 – low*

*10 – very satisfied*

- [scale from 1 to 10]

<u>Oncology – 25:</u>

What are the reasons for your dissatisfaction?

*Please select the applicable answer(s).*

*Please skip this question if you selected 9 or 10 (very satisfied) in the previous question.*

- lack of close monitoring
- too few cardiological consultations
- little dialogue/feedback for high-risk patients
- no clear statements/determinations on cardiotoxic risk
- other [free-text entry]

<u>Oncology – 26:</u>

Analogous to the various boards in a hospital, is there a regular cardio-oncology board at your institution?

If yes, how often does it take place?

*Please select one answer*

- no
- yes, daily
- yes, once a week
- yes, twice a month
- yes, monthly
- yes, once a quarter
- other [free-text entry]

<u>Oncology – 27:</u>

Do you consider cardio-oncology boards useful?

*Please select one answer.*

- yes, very useful for every patient
- yes, but only for high-risk patients
- no, the therapeutic consequence would be too small

<u>Oncology – 28:</u>

In your view, how can the care of cardio-oncology patients be improved?

*Please select the applicable answer(s).*

- simplification of the guidelines
- digital tools for risk stratification
- tools for better determination of cardiotoxicity
- closer communication with treating cardiologists
- regular interdisciplinary continuing education
- option to contact a cardio-oncology expert network for difficult questions
- tools for monitoring cardiac status
- other [free-text entry]

***Internal medicine***

<u>Internal medicine – 1:</u>

Where do you work?

*Please select one answer.*

- University hospital
- University hospital with a dedicated cardio-oncology unit
- academic teaching hospital
- maximum-care hospital
- primary or standard-care hospital

<u>Internal medicine – 2:</u>

On average, how many chemotherapy patients are referred to you per month for diagnostics/cardiological co-management

*referred per month?*

*Please enter a numeric value.*

- [free-text entry]

<u>Internal medicine – 3:</u>

What percentage of these patients falls into the tumour entities listed below?

*Please provide an approximate value for the proportion of the listed diseases among the total number of your cancer patients.*

- Breast cancer [percentage between 0 and 100]

- Prostate cancer [percentage between 0 and 100]
- Colorectal cancer [percentage between 0 and 100]
- Lung cancer [percentage between 0 and 100]
- Bladder cancer [percentage between 0 and 100]
- Kidney cancer [percentage between 0 and 100]
- Cancer of the oral cavity and pharynx [percentage between 0 and 100]
- Leukaemias [percentage between 0 and 100]
- Non-Hodgkin lymphomas [percentage between 0 and 100]
- other cancers [percentage between 0 and 100]

<u>Internal medicine – 4:</u>

What percentage are outpatients?

*Please provide an approximate value.*

- proportion of outpatients [percentage between 0 and 100]

<u>Internal medicine – 5:</u>

At which steps in the treatment of patients receiving potentially cardiotoxic chemotherapy are you involved?

*Please select the applicable answer(s).*

- cardiotoxic-risk stratification of chemotherapy
- cardiological diagnostics before chemotherapy
- cardiological diagnostics during chemotherapy
- cardiological diagnostics after chemotherapy
- initiation of therapy when cardiotoxicity has occurred
- adjustment of therapy when cardiotoxicity has occurred
- tumour boards

<u>Internal medicine – 6:</u>

Do you perform stratification of the potential cardiotoxic risk of chemotherapy?

*Please select one answer.*

- yes, on my own

- yes, but in collaboration with an interdisciplinary team
- no
- no, this is generally the task of the treating specialist oncologist/haematologist

Internal medicine – 7:

Does the cardiotoxic-risk assessment you perform influence the choice of the patients' chemotherapy?

*Please select one answer.*

- yes, I give specific recommendations for adjusting therapy on my own
- yes, if needed I consult the oncologist/gynaecologist and the cardiotoxic risk is jointly evaluated for each patient
- no, there is no communication at all

Internal medicine – 8:

Which parameters do you include in the assessment of cardiotoxic risk?

*Please select the applicable answer(s).*

- pre-existing cardiac disease (e.g. heart failure, coronary artery disease)
- age (>60 years)
- sex
- diabetes mellitus
- renal insufficiency
- obesity
- high-dose anthracycline chemotherapy
- additional radiotherapy with the heart in the radiation field
- HER2 inhibitors, proteasome inhibitors, VEGF therapy
- elevated cardiac biomarkers
- left ventricular function
- other [free-text entry]

Internal medicine – 9:

Which examinations do you routinely perform in these patients?

*Please select the applicable answer(s).*

- history
- ECG
- echocardiography
- ergometry
- laboratory: troponin T (TnT)
- laboratory: BNP
- laboratory parameters such as blood glucose, GFR, lipid profile
- other [free-text entry]

<u>Internal medicine – 10:</u>

Consider a patient receiving anthracycline chemotherapy who already has known heart failure.

How often is this patient referred to you?

*Please select the applicable answer(s).*

- before initiation of chemotherapy
- after every cycle of therapy
- after every second cycle of therapy
- after completion of chemotherapy
- 3 months after completion of therapy
- 6 months after completion of therapy
- 12 months after completion of therapy
- 24 months after completion of therapy
- in the event of acute cardiac symptoms
- other [free-text entry]

<u>Internal medicine – 11:</u>

For patients with suspected chemotherapy-related cardiotoxicity, are you involved in the further decision on continuing/adjusting chemotherapy?

*Please select one answer.*

- always

- occasionally
- yes, but only in exceptional cases
- no

<u>Internal medicine – 12:</u>

At which echocardiographic criteria do you assume relevant cardiotoxicity and communicate this?

*Please select one answer.*

- LVEF decline > 40% to a value below the reference limits
- LVEF decline > 30% to a value below the reference limits
- LVEF decline > 20% to a value below the reference limits
- LVEF decline > 10% to a value below the reference limits
- any LVEF decline represents cardiotoxicity

<u>Internal medicine – 13:</u>

In addition to LVEF measurement, do you routinely use echocardiographic strain analysis for cardiac follow-up after chemotherapy?

*Please select one answer.*

- yes
- no

<u>Internal medicine – 14:</u>

What are the reasons why strain analyses are not performed?

*Please select the applicable answer(s).*

- lack of technical equipment
- too time-consuming
- acoustic windows often insufficient
- therapeutic consequence unclear
- lack of expertise
- lack of personnel
- other [free-text entry]

Internal medicine – 15:

Do you measure NT-proBNP or troponin, and if so, in what percentage of treated patients did you yourself measure NT-proBNP or troponin before chemotherapy?

*Please select the applicable answer(s).*

*Please indicate, if applicable, the proportion of treated patients (in percent) in whom you yourself measured NT-proBNP or troponin before chemotherapy.*

- no, neither NT-proBNP nor troponin is measured
- yes, NT-proBNP is measured [free-text entry]
- yes, troponin is measured [free-text entry]

Internal medicine – 16:

Again consider a patient receiving anthracycline chemotherapy who already has known heart failure. How often do you measure these cardiac values?

*Please select the applicable answer(s).*

- before initiation of chemotherapy
- after every cycle of therapy
- after every second cycle of therapy
- after completion of chemotherapy
- 3 months after completion of therapy
- 6 months after completion of therapy
- 12 months after completion of therapy
- in the event of acute cardiac symptoms
- for: [free-text entry]
- no, no values are measured

Internal medicine – 17:

What are the reasons why these values are not measured?

*Please select the applicable answer(s).*

- This should be the task of the oncologist.
- costs too high
- The therapeutic consequence is unclear.

- little expertise in interpreting the results
- other [free-text entry]

<u>Internal medicine – 18:</u>

Do you know and use cardioprotective agents in the treatment of oncology patients?

*Please select one answer.*

- yes, I know and use cardioprotective agents in the treatment of oncology patients
- I know cardioprotective agents in the treatment of oncology patients but do not use any cardioprotective
- agents in the treatment of oncology patients
- no, I do not know or use any cardioprotective agents in the treatment of oncology patients

<u>Internal medicine – 19:</u>

Which agents do you know and use in your patients?

*Please select the applicable answer(s).*

- ACE inhibitors (e.g. enalapril)
- beta-blockers (e.g. carvedilol)
- ARNI (e.g. Entresto)
- aldosterone antagonists (e.g. spironolactone)
- statins (e.g. atorvastatin)
- iron chelators (e.g. dexrazoxane)
- antiplatelet agents (e.g. aspirin)
- anticoagulants (e.g. enoxaparin, rivaroxaban)
- neprilysin inhibitors
- other [free-text entry]

<u>Internal medicine – 20:</u>

Who usually initiates cardioprotective treatment in these patients?

*Please select one answer.*

- the treating oncologist
- the outpatient cardiologist

- the general practitioner
- myself
- I only formulate a recommendation
- other [free-text entry]

Internal medicine – 21:

Is there close dialogue between you and the treating oncologists regarding the results of cardiac diagnostics and their implications for the resulting therapy (both cardiological and oncological)?

*Please select one answer.*

- yes, there is always an active dialogue, if needed also by telephone, which also influences therapy
- yes, there is an active dialogue, if needed also by telephone, for certain high-risk patients, which also influences therapy
- no, I give specific therapeutic advice in the written report
- no, the treating oncologists/gynaecologists should interpret all essential information from the diagnostics
- themselves
- other [free-text entry]

Internal medicine – 22:

In your view, is specialised cardio-oncology care useful for chemotherapy patients?

*Please select one answer.*

*Please provide a reason, if applicable.*

- yes [free-text entry]
- no [free-text entry]

Internal medicine – 23:

Analogous to the various boards in a hospital, is there a regular cardio-oncology board at your institution?

If yes, how often does it take place?

*Please select one answer.*

*Please indicate the frequency, if applicable.*

- yes [free-text entry]

- no

Internal medicine – 24:

Do you consider cardio-oncology boards useful?

*Please select one answer*.

- yes, very useful for every patient
- yes, but only useful for high-risk patients
- no, the therapeutic consequence would be too small

Internal medicine – 25:

How do you rate the interdisciplinary care of cardio-oncology patients at your institution?

*Please indicate what percentage is missing to reach optimal care.*

- percentage to optimal care [percentage between 0 and 100]

Internal medicine – 26:

In your view, how can the care of cardio-oncology patients be improved?

*Please select the applicable answer(s).*

- simplification of the guidelines
- digital tools for risk stratification
- tools for better determination of cardiotoxicity
- closer communication with treating oncologists
- regular interdisciplinary continuing education
- option to contact a cardio-oncology expert network for difficult questions
- tools for monitoring cardiac status
- other
