# Supplementary material for: Cardio-oncology from the consulting side: diagnostic thresholds, surveillance and treatment responsibility among cardiologists and internists in a German multicentre survey
Source: Cardiooncology. 2026 Oct 7;12:123. doi: 10.1186/s40959-026-00588-7 (PMC13647822; doi:10.1186/s40959-026-00588-7)
Supplement: Supplementary file 2 [file 40959_2026_588_MOESM2_ESM.docx]

**Additional file 2. Mapping of survey items to analyses**

The table maps each thematic block of the anonymous online survey to the analysis in which it is reported and indicates whether it forms part of the present (secondary) analysis or of the companion reports. The division of items between the two hospital-based analyses was defined before analysis. As an exploratory descriptive survey, no single primary outcome was defined; the pre-specified objective of the present analysis was to characterise the cardiological contribution to the pathway (items marked “Present analysis”).

| **Survey item (code)** | **Content** | **Reported in** |
| --- | --- | --- |
| FA01 | Department / specialty | Companion report (participant characteristics) |
| KA01 | Type of institution | Companion report (participant characteristics) |
| KA02_01 / GY02_01 | Monthly patient volumes | Companion report (participant characteristics) |
| KA05 | Steps of the pathway in which cardiology/internal medicine are involved | Present analysis (Additional file 3: Fig. S1) |
| GY05_01 | Proportion under cardiological co-management | Companion report |
| GY08 | Steps at which treating specialties involve cardiologists | Companion report |
| KA06 / KA07 | Risk stratification by cardiology/internal medicine; influence on therapy | Present analysis (text) |
| GY11 | Influence of risk assessment on chemotherapy (treating specialties) | Companion report |
| GY12 | Investigations requested from cardiology | Present analysis (Table 1) |
| KA09 | Standard work-up performed by cardiology | Companion report |
| KA12 / GY16 | LVEF-decline thresholds for assuming cardiotoxicity | Present analysis (Fig. 1) |
| KA13 / KA14 | Routine strain use and barriers | Present analysis (text; Additional file 3: Table S1) |
| KA16 | Biomarker testing in the high-risk scenario | Present analysis (Table 2) |
| GY19 | Biomarker testing in the high-risk scenario (gynaecology/oncology) | Companion report |
| KA18 | Familiarity with and use of cardiovascular drugs | Present analysis (Additional file 3: Fig. S4) |
| KA19 / GY21 | Individual cardiovascular drugs used | Present analysis (Additional file 3: Table S3) |
| KA20 / IN20 / GY22 / ON22 | Physician who initiates cardioprotective therapy | Present analysis (Fig. 2) |
| KA21 | Interdisciplinary dialogue on cardiac findings | Present analysis (Additional file 3: Fig. S2) |
| GY14 | Interdisciplinary dialogue (treating specialties) | Companion report |
| KA23 / GY25 / KA24 | Cardio-oncology boards | Companion report |
| GY23_01 | Satisfaction with cardiological care (Likert) | Companion report |
| GY24 | Reasons for dissatisfaction | Present analysis (Additional file 3: Fig. S3) |

*Note: item codes reflect the survey’s internal labelling.*
