# Supplementary material for: Cardio-oncology from the consulting side: diagnostic thresholds, surveillance and treatment responsibility among cardiologists and internists in a German multicentre survey
Source: Cardiooncology. 2026 Oct 7;12:123. doi: 10.1186/s40959-026-00588-7 (PMC13647822; doi:10.1186/s40959-026-00588-7)
Supplement: Supplementary file 3 [file 40959_2026_588_MOESM3_ESM.docx]

**Additional file 3. Supplementary material**

*Cardio-oncology from the consulting side: diagnostic thresholds, surveillance and treatment responsibility among cardiologists and internists in a German multicentre survey.*

Contents: Supplementary Figures S1–S4 and Supplementary Tables S1–S3.

**Fig. S1 Involvement of internal medicine and cardiology across the cardio-oncology pathway.** Self-reported involvement of internal-medicine (n = 17) and cardiology (n = 35) respondents at successive steps of the potentially cardiotoxic treatment pathway. Multiple responses were possible.

**Fig. S2 Communication of cardiac findings to treating oncologists, cardiology versus internal medicine.** Mode of communication of cardiac-diagnostic findings and their therapeutic implications (cardiology n = 33, internal medicine n = 11). One response per respondent.

**Fig. S3 Reasons for dissatisfaction with cardio-oncology care, gynaecology versus oncology.** Reasons cited among dissatisfied respondents (gynaecology n = 18, oncology n = 24). Multiple responses were possible.

**Fig. S4 Familiarity with and use of cardiovascular drugs, by specialty.** Distribution of responses (“knows and uses”, “knows but does not use”, “neither”) to the single item on “cardioprotective substances”, by specialty (gynaecology n = 16, internal medicine n = 11, cardiology n = 33, oncology n = 23). The item combined primary prophylaxis, treatment of established dysfunction and conventional cardiovascular indications across a heterogeneous drug list; it does not measure guideline-defined cardioprotection and is presented descriptively only.

**Table S1 Reasons for not performing echocardiographic strain imaging, by discipline**

| **Reason (among non-users)** | **Cardiology** | **Internal medicine** |
| --- | --- | --- |
| Lack of expertise | 12.5 | 57.1 |
| Insufficient acoustic windows | 0 | 28.6 |
| Excessive time requirement | 50.0 | 14.3 |
| Lack of personnel | 50.0 | 14.3 |
| Lack of technical equipment | 25.0 | 14.3 |
| Unclear therapeutic consequence | 12.5 | 14.3 |

*Values are valid percent among respondents not using strain routinely (cardiology n = 8, internal medicine n = 7). These counts are very small and are shown for descriptive purposes only; multiple responses were possible.*

**Table S2 Item-level denominators and missing data**

| **Item (analysis)** | **Group** | **n – eligible to receive** | **n – answered** | **n – excluded by skip logic** | **n – excluded by non-response** |
| --- | --- | --- | --- | --- | --- |
| Involvement (Fig. S1) | Internal medicine | 21 | 17 | 0 | 4 |
| Involvement (Fig. S1) | Cardiology | 44 | 35 | 0 | 9 |
| Risk stratification | Cardiology + internal medicine | 65 | 51 | 0 | 14 |
| Influence on therapy | Cardiology + internal medicine | 65 | 26 | 25 | 14 |
| Requested work-up (Table 1) | Gynaecology | 27 | 20 | 0 | 7 |
| Requested work-up (Table 1) | Oncology | 28 | 24 | 0 | 4 |
| LVEF threshold (Fig. 1 – Panel A) | Cardiology + internal medicine | 65 | 47 | 0 | 18 |
| LVEF threshold (Fig. 1 – Panel B) | Gynaecology + oncology | 55 | 38 | 0 | 17 |
| Strain use | Cardiology + internal medicine | 65 | 45 | 0 | 20 |
| Strain barriers (Table S1) | Cardiology + internal medicine | 65 | 15 | 30 | 20 |
| Biomarker timing (Table 2) | Cardiology + internal medicine | 65 | 45 | 0 | 20 |
| Drug familiarity/use (Fig. S4) | All four specialties | 120 | 83 | 0 | 37 |
| Drugs used (Table S3) | Cardiology + internal medicine | 65 | 20 | 24 | 21 |
| Drugs used (Table S3) | Gynaecology + oncology | 55 | 18 | 25 | 12 |
| Initiator (Fig. 2) | Cardiology | 44 | 33 | 0 | 11 |
| Initiator (Fig. 2) | Internal medicine | 21 | 10 | 0 | 11 |
| Initiator (Fig. 2) | Gynaecology | 27 | 14 | 0 | 13 |
| Initiator (Fig. 2) | Oncology | 28 | 23 | 0 | 5 |
| Dialogue (Fig. S2) | Cardiology + internal medicine | 65 | 44 | 0 | 21 |
| Dissatisfaction (Fig. S3) | Gynaecology + oncology | 55 | 42 | 0 | 13 |

For every table and figure in the manuscript and this supplement, the table reports the number of respondents eligible to receive the item — those to whom it was displayed given specialty and preceding skip logic — the number who answered it, the number excluded by skip logic and the number lost to item non-response. Eligible n equals answered plus skip-excluded plus non-response. Percentages in the main text and figures use the number answering as the denominator.

**Table S3 Cardiovascular (“cardioprotective”) drugs, by specialty and indication**

| **Agent** | **Cardiology** | **Internal medicine** | **Gynaecology** | **Oncology** |
| --- | --- | --- | --- | --- |
| Neurohormonal agents |  |  |  |  |
| ACE inhibitor | 93.8 | 100 | 25 | 78.6 |
| ARNI | 68.8 | 100 | – | – |
| Beta-blocker | 87.5 | 100 | 25 | 78.6 |
| MRA | 75.0 | 100 | 0 | 57.1 |
| Potentially cardioprotective agents |  |  |  |  |
| Statin | 75.0 | 75.0 | 25 | 50.0 |

*Values are valid percent among item-specific valid responders (cardiology n = 16, internal medicine n = 4, gynaecology n = 4, oncology n = 14). ARNI was not offered as a response option to gynaecology or oncology (“–”); the internal-medicine and gynaecology columns rest on very few respondents and are shown for completeness. Multiple responses were possible. ACE, angiotensin-converting enzyme; ARNI, angiotensin receptor–neprilysin inhibitor; MRA, mineralocorticoid-receptor antagonist.* Antiplatelet agents, anticoagulants and iron chelators were offered as response options but are not reported here: the first two are not cardioprotective with respect to cancer therapy-related cardiac dysfunction, and the item wording did not distinguish this narrow sense of cardioprotection from the broader prevention of cardiovascular complications of cancer therapy. Because the response options were not identical across specialties, between-specialty comparison of this table is not valid for any agent.
